# The Thyrotropin-Releasing Hormone-Degrading Ectoenzyme, a Therapeutic Target?

**DOI:** 10.3389/fphar.2020.00640

**Published:** 2020-05-08

**Authors:** Jean-Louis Charli, Adair Rodríguez-Rodríguez, Karina Hernández-Ortega, Antonieta Cote-Vélez, Rosa María Uribe, Lorraine Jaimes-Hoy, Patricia Joseph-Bravo

**Affiliations:** Departamento de Genética del Desarrollo y Fisiología Molecular, Instituto de Biotecnología, Universidad Nacional Autónoma de México (UNAM), Cuernavaca, Mexico

**Keywords:** thyroid hormone, thyrotropin, thyrotropin-releasing hormone, thyrotropin-releasing hormone-degrading ectoenzyme, anxiety, depression, mood

## Abstract

Thyrotropin releasing hormone (TRH: Glp-His-Pro-NH_2_) is a peptide mainly produced by brain neurons. In mammals, hypophysiotropic TRH neurons of the paraventricular nucleus of the hypothalamus integrate metabolic information and drive the secretion of thyrotropin from the anterior pituitary, and thus the activity of the thyroid axis. Other hypothalamic or extrahypothalamic TRH neurons have less understood functions although pharmacological studies have shown that TRH has multiple central effects, such as promoting arousal, anorexia and anxiolysis, as well as controlling gastric, cardiac and respiratory autonomic functions. Two G-protein-coupled TRH receptors (TRH-R1 and TRH-R2) transduce TRH effects in some mammals although humans lack TRH-R2. TRH effects are of short duration, in part because the peptide is hydrolyzed in blood and extracellular space by a M1 family metallopeptidase, the TRH-degrading ectoenzyme (TRH-DE), also called pyroglutamyl peptidase II. TRH-DE is enriched in various brain regions but is also expressed in peripheral tissues including the anterior pituitary and the liver, which secretes a soluble form into blood. Among the M1 metallopeptidases, TRH-DE is the only member with a very narrow specificity; its best characterized biological substrate is TRH, making it a target for the specific manipulation of TRH activity. Two other substrates of TRH-DE, Glp-Phe-Pro-NH_2_ and Glp-Tyr-Pro-NH_2,_ are also present in many tissues. Analogs of TRH resistant to hydrolysis by TRH-DE have prolonged central efficiency. Structure-activity studies allowed the identification of residues critical for activity and specificity. Research with specific inhibitors has confirmed that TRH-DE controls TRH actions. TRH-DE expression by β2-tanycytes of the median eminence of the hypothalamus allows the control of TRH flux into the hypothalamus-pituitary portal vessels and may regulate serum thyrotropin secretion. In this review we describe the critical evidences that suggest that modification of TRH-DE activity in tanycytes, and/or in other brain regions, may generate beneficial consequences in some central and metabolic disorders and identify potential drawbacks and missing information needed to test these hypotheses.

## Introduction

Thyrotropin releasing hormone (TRH; Glp-His-Pro-NH_2_) is a small peptide expressed mainly in the brain, secreted by neurons. TRH interacts with plasma membrane G-protein-coupled receptors; one TRH receptor (TRH-R1) is detected in humans, whereas an additional one (TRH-R2) in rodents (Sun et al., 2003) and a total of three in teleosts and frogs (Bidaud et al., 2004; Saito et al., 2011). In mammals, TRH-R1 and TRH-R2 arise from different genes (Sun et al., 2003); the maps of their brain expression do not coincide (Heuer et al., 2000); they possess distinct ligand-independent activities, with TRH-R2 showing a stronger constitutive activity (Wang and Gershengorn, 1999). These distinct characteristics and the differential behaviors of KO mice for each receptor (Rabeler et al., 2004; Zeng et al., 2007; Sun et al., 2009) suggest that each type of receptor fulfills distinct functions in response to TRH. However, data in mice indicate that central TRH effects may depend mainly on TRH-R1 expression (Thirunarayanan et al., 2013).

The best-known function of TRH is the control of the hypothalamus–pituitary–thyroid (HPT) axis; TRH is synthesized by processing of a protein precursor in neurons of the paraventricular nucleus of the hypothalamus (PVN) that project their terminal boutons into the median eminence (hypophysiotropic neurons). Once released into the extracellular space TRH diffuses into the hypothalamic-pituitary portal vessels from where it controls secretion of thyrotropin (TSH) from the anterior pituitary. In turn, TSH controls the synthesis and secretion of thyroid hormones (TH) in the thyroid. This link adjusts TH secretion according to energy balance information, which is integrated by the hypophysiotropic PVN TRH neurons. TRH may also control prolactin secretion from the anterior pituitary (Martinez de la Escalera and Weiner, 1992; Joseph-Bravo et al., 2015b), although hypophysiotropic PVN TRH neurons regulating prolactin may differ from those controlling TSH secretion (Sánchez et al., 2001).

Various additional hypothalamic and extrahypothalamic neuronal populations express *Trh* (Lechan et al., 1986; Segerson et al., 1987; Hökfelt et al., 1989; Heuer et al., 2000), but their physiological role is not well understood. Soon after the discovery of extrahypothalamic TRH, several studies revealed central pharmacological effects of TRH that were independent of HPT axis control in laboratory animals, including increased locomotion, arousal, improvement of depression-like behaviors, reduction of epileptic seizures, and neuroprotection (Horita, 1998; Gary et al., 2003; Daimon et al., 2013; Fröhlich and Wahl, 2019). These observations led to attempts to use TRH in therapeutic contexts, for example in some neurodegenerative diseases (Gary et al., 2003; Daimon et al., 2013; Fröhlich and Wahl, 2019). Moreover, TRH is also present in selected loci outside the central nervous system whose importance is still poorly appreciated.

Early evidence that the half-life of TRH in rodent blood or in tissue extracts is 3–5 min (Redding and Schally, 1969; Prasad and Peterkofsky, 1976; Taylor and Dixon, 1976), and that TRH pharmacological effects are of short duration led to the synthesis of TRH analogues with improved stability, agonist potency and/or brain accessibility (Horita, 1998; Gary et al., 2003; Daimon et al., 2013; Fröhlich and Wahl, 2019). Other efforts were directed to elucidate the mechanism contributing to the rapid extracellular disappearance of TRH. TRH can be removed from the brain extracellular space by transport into brain cells; nevertheless, this event has a small V_max_ and may have a very limited quantitative importance (Charli et al., 1984). The molecular entity that contributes to this transport has not been characterized; it may reflect TRH-R mediated endocytosis (Ashworth et al., 1995), or the action of a TRH transporter (Bagul et al., 2014).

Alternatively, TRH may be hydrolyzed by peptidases. Pyroglutamyl peptidase I (PPI; EC 3.4.19.3) is a soluble cysteine aminopeptidase with a wide specificity, that hydrolyses almost any Glp-X peptide, except if X is a proline (Awadé et al., 1994). This enzyme is present in all life kingdoms, and found in many tissues, including brain. Although PPI hydrolyses TRH *in vitro* (Morier et al., 1979; O'Leary and O'Connor, 1995; Charli et al., 1998), the only evidence that it contributes to TRH turnover *in situ* is that an inhibitor of PPI (and PE) enhances TRH levels and release in primary cultures of hypothalamic cells (Faivre-Bauman et al., 1986). Since PPI action is probably restricted to the cytoplasm, it may contribute to the intracellular turnover of TRH leaking from intracellular granules, but its subcellular localization is not compatible with a post-secretory role. In support of this idea, the specific inhibition of PPI activity in brain slices does not change TRH release and the intraperitoneal injection of a specific inhibitor of PPI that inhibits brain PPI activity does not change brain TRH levels (Charli et al., 1987). Furthermore, inhibition of enzyme activity does not protect TRH from degradation in rat serum (Friedman and Wilk, 1985).

Prolyl endopeptidase (PE; EC 3.4.21.26) is a serine endopeptidase with a wide specificity; it hydrolyses internal Pro-X peptide bonds, except if X is a proline. This enzyme is soluble, present in all life kingdoms, and many tissues, including the mammalian brain, albeit a membrane-bound isoform has also been identified. PE hydrolyzes the Pro-NH_2_ bond of TRH *in vitro* (Wilk, 1983; O'Leary and O'Connor, 1995; Charli et al., 1998; Tenorio-Laranga et al., 2008). PE role in TRH turnover *in vivo* is controversial; inhibition of PE enhances TRH levels and release in primary cultures of hypothalamic cells (Faivre-Bauman et al., 1986), the intraperitoneal injection of a PE inhibitor enhances TRH concentration in the cerebral cortex (Bellemere et al., 2005), and PE or a similar enzyme degrades TRH in rabbit seminal plasma (Siviter and Cockle, 1995). However, other experiments showed that PE inhibition in brain slices does not change TRH release into the medium and that the intraperitoneal injection of a specific inhibitor of PE does not change brain TRH levels *in vivo* (Charli et al., 1987). Furthermore, the intraseptal injection of a PE inhibitor does not change the effect of TRH on time of arousal from ethanol-induced narcosis in rats (Lazcano et al., 2012). Since PE regulates inositol polyphosphate phosphatase activities (Williams et al., 1999), the effect of PE inhibition on TRH levels may result from an alteration of the intracellular turnover of the secretory granules that contain TRH. Likewise, inhibition of PE does not protect TRH from degradation in rat serum (Friedman and Wilk, 1985). Thus, in general, PE may not control the extracellular turnover of TRH.

Another peptidase that may hydrolyze TRH is prolyl carboxypeptidase (PCP; EC 3.4.16.2) (Jeong et al., 2012), but there is no evidence that PCP coincides with TRH or alters TRH biological actions *in vivo*. Therefore, the quantitative importance of the previously described mechanisms of inactivation of TRH may be very limited *in vivo*, in particular for its extracellular inactivation, due in most cases to incompatible subcellular localizations of peptide and enzymes (Charli et al., 1998). In sum, contrary to what is sometimes mentioned in the literature, neither uptake nor soluble peptidases constitute relevant mechanisms for the extracellular inactivation of TRH.

The discovery of the thyrotropin-releasing hormone-degrading ectoenzyme (TRH-DE; EC 3.4.19.6) stems from the identification of an enzymatic activity that hydrolyses the Glp–His bond of TRH, initially in serum and called thyroliberinase, to underpin its narrow specificity (Bauer et al., 1981), and later in brain (Garat et al., 1985). The most striking property of this pyroglutamyl peptidase is a very narrow specificity (Lanzara et al., 1989; Wilk and Wilk, 1989; Elmore et al., 1990; Gallagher and O'Connor, 1998; Heuer et al., 1998a), a compelling argument in favor of looking at this peptidase as a therapeutic target, for example when enhancement of TRH action may be beneficial. TRH-DE was initially named pyroglutamyl peptidase II (PPII), to stress its clear biochemical difference with PPI; in contrast to PPI, TRH-DE/PPII is a metallopeptidase (Czekay and Bauer, 1993). A review describing the major biochemical characteristics of TRH-DE was previously published (Sánchez et al., 2013).

In the next sections, we briefly review the structure and proposed physiological roles of TRH-DE, focusing on recent advances, and we identify major challenges to translate this knowledge to clinical applications. Although the biochemical, topological and functional properties of the enzyme strongly argue in favor of its implication in the extracellular inactivation of TRH, its role may vary with cellular context, and might extend beyond hydrolysis of TRH. Contexts in which an intervention on TRH-DE may have therapeutic potential are delineated, and limitations are identified.

## *Trhde* Gene, Transcripts, Protein Structure, Isoforms and Specificity

The sequence and genomic locus of the *Trhde* gene (we use the rodent abbreviations for gene names throughout the review) was initially described in humans (Schomburg et al., 1999); there is only one gene localized on chromosome 12. In mammals, the *Trhde* gene is adjacent to the tryptophan hydroxylase 2 gene (https://www.ensembl.org). In mouse, the gene localizes to chromosome 10 (http://www.informatics.jax.org/marker/MGI:2384311) while in rats, to chromosome 7. *Trhde* gene sequences and enzymatic activities have been detected in mammals, including activity in human serum and cerebrospinal fluid (Prasad and Jayaraman, 1986; Bundgaard and Møss, 1990; Charli et al., 1998). The gene is also detected in other vertebrate classes (Schomburg et al., 1999; Chávez-Gutiérrez et al., 2006). TRH-DE is not an essential gene in mice as TRH-DE KO animals are healthy, reproduce normally and their metabolic parameters are normal when bred in standard conditions (Tang et al., 2010).

**Figure 1:**
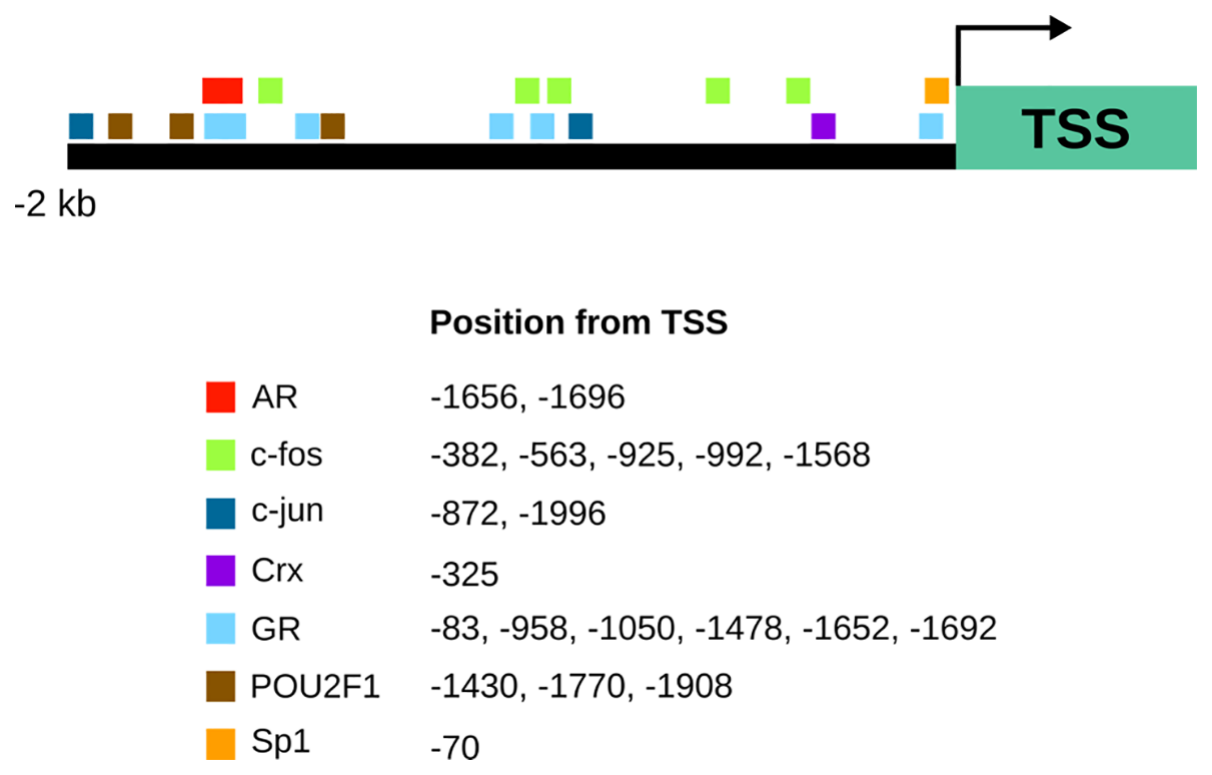
A map of candidates for transcriptional regulation of *Trhde* expression predicted by bioinformatic analysis. Transcription factor binding sites were identified 2 kb upstream of the most upstream of the 3 predicted transcriptional start sites (TSSR99301) of the mouse with PROMO (Messeguer et al., 2002; Farre, 2003). This site is conserved in rats. AR, androgen receptor; Crx, Cone-rod homeobox protein; GR, glucocorticoid receptor; NF-kappaB, nuclear factor kappa-light-chain-enhancer of activated B cells; POU2F1, POU domain, class 2, transcription factor 1; Sp1, specificity protein 1.

*Trhde* transcription initiation sites have not been formally mapped. In mouse, three transcription start sites have been proposed (http://www.informatics.jax.org/marker/MGI:2384311); two are conserved in rat for which various transcripts differing widely in size have been detected (Schauder et al., 1994). Apart from alternative transcription start sites, there is little information about the mechanisms that lead to transcript diversity, except for a potential alternative splicing event in rats at exon 14-intron 14 boundary that leads to a truncated TRH-DE isoform (Chavez-Gutierrez et al., 2005). In mice three alternative splicing events may generate various TRH-DE isoforms (http://www.informatics.jax.org/). Bioinformatic approaches suggest that in mice response elements for multiple transcription factors exist along the 2 Kb region upstream from the most upstream of the predicted transcriptional start sites (**Figure 1**). However, promoter structure and genomic regulatory elements have not been experimentally mapped.

Another transcript derived from the human *Trhde* locus is *Trhde* antisense RNA 1 (*Trhde-as1*) (https://www.ncbi.nlm.nih.gov/nuccore/NR_026837). This long non-coding RNA is found in the cytoplasm and binds directly to miR-103 in samples of human lung, inhibiting parameters of human lung cancer progression (Zhuan et al., 2019). Individuals challenged with an injection of LPS show a significant drop of *Trhde-as1* expression in adipose tissue samples (Ferguson et al., 2016). The relevance of *Trhde-as1* expression for *Trhde* function is unknown.

The purification and cloning of the rat brain/pituitary isoform revealed that TRH-DE is 1,025 amino acids long (1,024 in human) (Bauer, 1994; Schauder et al., 1994). TRH-DE is a member of the M1 family of zinc-aminopeptidases, a family of 12 members (in humans) that includes aminopeptidases that have a wider specificity than TRH-DE, including aminopeptidase N (the closest relative of TRH-DE), which preferentially hydrolyses neutral aminoacids from the N-terminus (Sjöström et al., 2002). Although TRH-DE 3D structure is still unknown, analogy with other members of the family suggests it is a type II cell surface peptidase with a small intracellular domain, a single transmembrane domain, and a large extracellular part with a flexible stem followed by a catalytic domain, and an additional domain separated by a flexible loop (Chávez-Gutiérrez et al., 2006). Topology of the domains is consistent with biochemical evidence that demonstrated TRH-DE is an ectoenzyme (Charli et al., 1988), making it a prime candidate for primary hydrolysis of TRH in the extracellular space.

A large set of TRH derivatives has been tested to define TRH-DE specificity; TRH analogues modified on the C- or N-terminal residues are poor substrates of TRH-DE (Lanzara et al., 1989; Wilk and Wilk, 1989; Heuer et al., 1998a; Kelly et al., 2000). For example, gonadotropin-releasing hormone (GnRH), a decapeptide with a N-terminal Glp-His is not a substrate of TRH-DE (O'Connor and O'Cuinn, 1985; Elmore et al., 1990; Gallagher and O'Connor, 1998). Substitution of the histidyl residue of TRH by a series of amino acids influences the ability of porcine TRH-DE to catalyze the hydrolysis of the Glp-X bond in TRH-like peptides; thus, TRH-like peptides substituted with Gly, Asn, Gln, Trp, Glu, Asp, Pro or Cys are not substrates of TRH-DE, while low turnover rates are observed with Ala, Ser, Thr, Ile, Leu, Val. In contrast, TRH-like peptides substituted with Tyr or Phe have turnover rates approaching that of TRH (Kelly et al., 2000). This agrees with evidence that Glp-Phe-Pro-NH_2_ is a substrate of bovine brain TRH-DE (Kelly et al., 1997; Gallagher and O'Connor, 1998). The structural determinants of TRH-DE narrow specificity have been partially clarified; two amino-acid substitutions in the catalytic domain are critical and form a conserved signature that distinguishes it from other M1 family members (Chávez-Gutiérrez et al., 2006).

One of the two TRH-like peptides that are relatively good substrates of the enzyme *in vitro*, Gln-Phe-Pro-NH_2_, has been detected in peripheral organs including pancreas and thyroid gland (Khan et al., 1992; Gkonos et al., 1994; Kulkarni et al., 1995). Although it was initially suggested that TRH-like peptides, other than TRH, were not detectable in brain (Rondeel et al., 1995a; Rondeel et al., 1995b), it was later shown that, among other, Gln-Tyr-Pro-NH_2_, and Gln-Phe-Pro-NH_2_ are present in brain (Pekary et al., 2004; Pekary et al., 2005). To our knowledge, although these peptides are likely the product of posttranslational processing of precursor peptides (Linden et al., 1996), the precursors are still unknown. However, they may act as intercellular messengers, including in the brain, since their concentrations can vary rapidly in response to some stimuli, suggesting changes in synthesis and/or release (Pekary and Sattin, 2012; Pekary et al., 2015), and there is direct evidence that a peptide similar to Gln-Phe-Pro-NH_2_ can be released into hypothalamic extracellular space *in vitro* (Méndez et al., 1999). *Bona fide* receptors for Gln-Phe-Pro-NH_2_ and Gln-Tyr-Pro-NH_2_ have not been detected; they are weak agonists of TRH receptors (Hinkle et al., 2002).

Information about TRH-DE biosynthesis, transport and turnover is scant. The membrane isoform is a N-glycosylated protein; within its large extracellular domain, 12 putative N-glycosylation sites are found (Schauder et al., 1994). In its native form, TRH-DE can be partially deglycosylated (by about 50%) without affecting its activity (Bauer, 1994). The relevance of remaining glycosyl residues is not yet known. N-linked protein glycosylation starts in the endoplasmic reticulum and is a conserved process in eukaryotic cells. The subsequent details of processing that occur in Golgi compartments, according to protein-, cell- and species-specific clues (Aebi, 2013), have not been described for TRH-DE. Thyroliberinase, the soluble isoform secreted into blood, may be a product of alternative splicing or proteolysis (Schmitmeier et al., 2002). A membrane-bound truncated TRH-DE (TRH-DE*) is detected in many tissues, formed probably through an alternative splicing event; TRH-DE* does not hydrolyze TRH but it inhibits TRH-DE activity when it dimerizes with the full-length TRH-DE (Chavez-Gutierrez et al., 2005). In line with these results, correlative evidence suggests that the tissue-specific pattern of TRH-DE activity is defined in part by the *Trhde*/Trhde* expression ratio (Chavez-Gutierrez et al., 2005). Finally, little is known about TRH-DE interactions with other proteins. TRH-DE* may interact physically with Akt1 in mesenchymal-derived soft tissue sarcoma (STS) cell lines (Zhu et al., 2011) but an interpretation of the physiological relevance of this interaction awaits resolution.

## Specific Inhibitors of TRH-DE

To investigate the biological functions of TRH-DE, TRH-DE inhibitors have been synthesized. Protease inhibitors frequently include structural features that enable their interaction with the active site of the enzyme (Cushman et al., 1977; Kaysser, 2019). TRH-DE is weakly inhibited by the products of hydrolysis of TRH, Glp and His-Pro-NH_2_; combination of these molecules with zinc-binding groups results in poor TRH-DE inhibitors (Bauer et al., 1997). In general, TRH derivatives with modifications to the C- or N-terminal residues of TRH do not inhibit TRH-DE activity (Lanzara et al., 1989; Wilk and Wilk, 1989; Elmore et al., 1990; Gallagher and O'Connor, 1998; Kelly et al., 2000). However, GnRH, a Glp-His peptide C-terminally extended with hydrophobic residues does inhibit TRH-DE activity (O'Connor & O'Cuinn, 1985; Elmore et al., 1990; Gallagher and O'Connor, 1998). Furthermore, some analogues modified on the histidine residue have also shown some potential. Thus, the first specific inhibitor described was N-[1-carboxy-2-phenylethyl]N-imidazole benzyl histidyl-β-naphthylamide (CPHNA). With a Ki of 8 µM, CPHNA has no effect on TRH receptors and does not inhibit PE activity (Charli et al., 1989).

The systematic screening of a directed peptide library led to the finding that Glp-Asn-Pro-7-amido-4-methyl coumarin (Glp-Asn-Pro-AMC) has a Ki of 0.97 µM, making this peptide a relatively potent reversible TRH-DE inhibitor (Kelly et al., 2000). Improved potency was achieved by extension of Glp-Asn-ProNH_2_ with hydrophobic amino acids at the C-terminus. Thus, Glp-Asn-Pro-Tyr-Trp-Trp-AMC displays a Ki of 1 nM, which makes it the most potent competitive TRH-DE inhibitor described to date. Unfortunately, the *in vivo* potency of this peptide is poor (Kelly et al., 2005)

Another potent synthetic inhibitor of TRH-DE is the phosphinic analogue of TRH, GlpΨ[P(O)(OH)]HisProNH2 (Ψ-TRH) with a Ki of 170 nM, in which the scissile peptide bond of TRH has been replaced by the chemically stable phosphinic bond (Matziari et al., 2008).

Finally, a natural TRH-DE inhibitor named *Hermodice carunculata* protease inhibitor (HcPI) was isolated from the marine annelide *H. carunculata*. HcPI inhibits rat TRH-DE (a brain membrane extract with 7.5 mg protein/ml) with an IC_50_ of 4.8 μg/ml (Pascual et al., 2004). It is a small hydrophilic molecule very specific for TRH-DE, since it is at least 100 fold less potent at inhibiting the activity of the nearest homologue in the M1 family, aminopeptidase N (Cruz et al., 2008), and it is brain permeant (Pascual et al., 2004). HcPI structure is still unknown; solving it may lead to new tools.

## TRH Analogues Resistant to Hydrolysis

TRH is a poor drug candidate. Although TRH is stable in the gastrointestinal tract, it has low intestinal permeability (Yokohama et al., 1984). The intranasal route of delivery presents a possible alternative route to access the brain (Veronesi et al., 2007; Zada et al., 2019). Furthermore, TRH has also a short plasma half-life, being rapidly hydrolyzed by peptidases (Redding and Schally, 1969; Prasad and Peterkofsky, 1976; Taylor and Dixon, 1976), and its cerebral absorption is low (Cornford et al., 1978). Finally, TRH has extensive neuroendocrine, central and autonomic/cardiac effects that may limit its therapeutic use (Gary et al., 2013; Khomane et al., 2011; Joseph-Bravo et al., 2015b).

Thus, TRH analogues have been developed to increase stability, delivery, specificity and potency. The properties of these analogs have been previously described extensively (Colson and Gershengorn, 2006; Monga et al., 2008; Khomane et al., 2011; Gary et al., 2003). Their stability in the presence of TRH-DE has seldom been directly tested or was tested against tissue homogenates that contain irrelevant peptidases. However, their stability in serum is a relatively good measure of their resistance to TRH-DE, since thyroliberinase is the main activity degrading TRH in serum. In this section, we describe TRH analogues that are resistant to TRH-DE.

TRH-like peptides in which the N-terminal Glp residue is replaced with a non-natural amino acid have promising pharmacological properties. Taltirelin ([1-methyl-(S)-4,5-dihydroorotyl)]-L-His-L-ProNH_2_) was synthesized by replacing the Glp residue of TRH with (S)-4,5, dihydroorotic acid (Suzuki et al., 1990). Taltirelin produces central nervous system (CNS) effects at about 100 times lower doses than TRH and has eight times longer duration of antagonistic action on pentobarbital-induced sleep than TRH (Yamamura et al., 1990; Yamamura et al., 1991a; Kinoshita et al., 1998). The differences in the activities of Taltirelin and TRH in the CNS have been attributed to the higher stability of Taltirelin in blood against thyroliberinase, and its higher lipophilicity than TRH that account for its increased penetration across the blood–brain barrier. Taltirelin resistance to hydrolysis by TRH-DE has been questioned, as Taltirelin was rapidly degraded in rat cerebral and pituitary homogenates (Kodama et al., 1997; Kobayashi et al., 2019a), but it is likely that in these homogenates enzymes distinct from TRH-DE were actively degrading Taltirelin.

Montirelin (6-methyl-5-oxothiomorpholinyl-3-carbonyl-His-Pro-NH_2_, CG-3703) is a derivative of the Gln moiety of TRH, which is an efficient CNS-stimulating agent (Horita, 1998). This TRH analog readily penetrates into the brain after its systemic administration in rats (Itoh et al., 1995) and inhibits specific Gln-[3methyl-His]-Pro-NH2 binding (Ki = 35 nM) to rat brain receptors as efficiently as TRH (Ki = 39.7 nM) (Urayama et al., 2001). Montirelin exhibits high stability in the blood (Sugimoto et al., 1996) and shows strong resistance to enzymatic degradation by pyroglutamyl aminopeptidase from brain or other related pyroglutamate aminopeptidases from liver and pituitary (Bauer, 1979; Griffiths et al., 1982).

Azetirelin (N^α^-[[(5)-4-oxo2-azetidinyl]carbonyl]-His-Pro-NH_2_ dehydrate) has arousal actions 10–40 fold more potent and 8–36 times longer lasting than TRH (Yamamoto and Shimizu, 1987). This longer duration of action is probably because Azetirelin has a longer half-life in dogs and humans than TRH. However, Azetirelin has low oral bioavailability (Sasaki et al., 1994) and methods to improve its absorption have been evaluated (Sasaki et al., 1999).

Posatirelin (L-6-ketopiperidine-2-carbonyl-L-leucyl-proline amide) is a neutral analog with improved CNS activity and with cholinergic, catecholaminergic and neurotrophic properties (Oka et al., 1989; Amenta et al., 1997; Monga et al., 2008). Posatirelin has a stronger anticataleptic effect than TRH (Szirtes et al., 1986), and improves cognitive and motor disturbances in rats (Drago et al., 1996), while having poor influence on TSH release (Szirtes et al., 1984). Several human studies have also suggested beneficial effects in subjects with cognitive impairments (Cucinotta et al., 1994; Parnetti et al., 1995). Posatirelin is stable in human and rabbit serum, while it is slowly degraded in rat and mouse plasma (Terauchi et al., 1988).

Rovatirelin (1-{N-[(4S,5S)-(5-methyl-2-oxooxazolidin-4-yl)-carbonyl]-3-(thiazol-4-yl)-L-alanyl}-(2R)-2-methylpyrrolidine trihydrate) has a higher affinity for TRH-R1 (Ki = 702 nM) than Taltirelin (Ki = 3,880 nM) but still lower than TRH (Ki = 128 nM) in cell membrane preparation from CHO-K1 cells overexpressing the human TRH receptor (Ijiro et al., 2015). Rovatirelin is resistant to thyroliberinase in rat plasma and also to TRH-DE in pituitary (Kobayashi et al., 2018; Kobayashi et al., 2019a; Kobayshi et al., 2019b).

TRH-like peptides with simultaneous replacement of Glp by a panel of hetero ring-containing carboxylic acids and central histidine by 1-alkyl-L-histidines activate TRH-R2 with higher potencies than TRH-R1. One of these, with simultaneous substitution of Glp by pyrazine-2-carboxylic acid (2-Pyz) and central His with C_3_H_7_ (2-Pyz-_L_-His(1-alkyl)-_L_-Pro-NH_2_), exhibits high selectivity towards TRH-R2 but poor potency compared to TRH, high stability in rat blood plasma, antagonizes pentobarbital-induced sleeping time with a higher potency than TRH, and is devoid of adverse cardiovascular and CNS effects (Meena et al., 2015).

Another class of TRH derivative corresponds to those that have dual pharmacological activity, acting both as inhibitor of TRH-DE and as receptor agonist. Glp-Asn-Pro-AMC reversibly inhibits TRH-DE activity and binds preferentially to central TRH receptors (Kelly et al., 2000). The replacement of the hydrophobic L-amino acid residues with their D-isomers in C-terminally extended analogs of Glp-Asn-Pro-NH_2_ led to Glp-Asn-Pro-D-Tyr-D-Trp-NH_2_ (named JAK4D), which is effective at producing and potentiating some central actions of TRH without evoking TSH release *in vivo*. This peptide has high plasma stability and combined potent inhibition of TRH-DE (Ki 151 nM) with high affinity binding to central TRH receptors (Ki 6.8 nM). In addition, JAK4D has a high ability to cross the blood–brain barrier and shows a clean preliminary toxicology profile (Scalabrino et al., 2007; Kelly et al., 2015).

To conclude, TRH analogs incorporating resistance to hydrolysis by TRH-DE and/or inhibition of TRH-DE activity associate with enhanced pharmacological activity, compared to TRH.

## TRH-DE is Critical for the Extracellular Catabolism of TRH in the Central Nervous System

**Figure 2:**
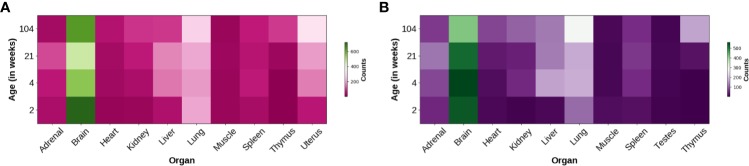
Distribution of *Trhde* expression in multiple rat organs. Heatmaps show the heterogeneous *Trhde* expression, predominant in the brain. Other differences can be observed along multiple developmental stages and between females **(A)** and males **(B)**. Data were refined from a database obtained from a transcriptomic profiling study in Fischer 344 rats (Yu et al., 2014; Hicks et al., 2018).

*Trhde* expression is detected at relatively late phases of brain development. TRH-DE activity appears in the male rat brain a few days before birth, and peaks at post-natal day 8 in hypothalamus and posterior cerebral cortex, while it decreases afterwards to adult values. The pattern differs in the olfactory bulb, two peaks of specific activity being observed at the 3th and 22nd day (Vargas et al., 1992b). *Trhde* expression is at least 10-fold higher in brain than in nine other organs of male or female Fisher 344 rats and this ratio is stable from 2 to 104 weeks (Yu et al., 2014) (**Figure 2**). Thus, the brain is the region with the highest TRH-DE specific activity in adult animals (Vargas et al., 1992a; Lin and Wilk, 1998).

*Trhde* transcript levels and activity vary widely among brain regions, with highest values detected in cerebral cortex and hippocampus, as well as in cerebellar hemispheres, regions with limited *Trh* expression (Vargas et al., 1987; Vargas et al., 1992a; Heuer et al., 1998b; Lin and Wilk, 1998). In the adult rat brain, the map of *Trhde* transcript distribution is generally consistent with the proposal that it is expressed by neurons (Heuer et al., 1998b). This is congruent with evidence that TRH-DE activity is enriched in synaptosomes (Torres et al., 1986) and that in primary culture of E17 hypothalamic cells, neurons are the major cell type expressing TRH-DE activity (Cruz et al., 1991). Furthermore, many rat cortical *Trhde* mRNA positive cells are *Slc17a7* mRNA positive (express vesicular glutamate transporter 1), and some *Gad* mRNA positive (express glutamic acid decarboxylase), which directly confirms that neurons express *Trhde* (Rodríguez-Molina et al., 2014). Finally, single cell transcriptomic data of mouse CNS and peripheral nervous system (Zeisel et al., 2018) (**Table 1**) indicate that apart from many neuron types, the majority of non-neuronal cells have undetectable levels of *Trhde* mRNA.

**Table 1:** Cells types along the mouse central and peripheral nervous system with the highest expression of *Trhde*, and relative expression of *Trhr* and *Trhr2*.

Region	Phenotype	*Trhde*	*Trhr*	*Trhr2*
Hippocamposeptal projection, cortex/hippocampus	GABA/Sst	+++++	−	++
Nucleus of the solitary tract	GABA/Gly/Ach	+++++	+	−
Myenteric plexus of small intestine	Ach	++++	−	−
Subiculum/Cortex	Glu	++++	+	++++
Hippocampus CA3	Glu	+++	+	−
Lateral cortex layer 6: gustatory, barrel field, auditory	Glu	+++	−	−
Superior Coliculus	Glu	++	−	−
Cortical pyramidal layer 4	Glu	++	−	−
Hippocampus interneurons	GABA	++	−	−
Interneuron-selective interneurons, cortex/hippocampus	GABA	++	−	−
Cortical pyramidal layer 6b	Glu	++	−	−
Cortical pyramidal layer 6	Glu	++	−	−
Entorhinal superficial layers	Glu	++	−	−
Spinal cord, Dorsal cord lamina 2-5	Glu	++	−	−
Inhibitory neurons, hindbrain	GABA/Gly	++	+	++
Septal nucleus, Meissnert and diagonal band	GABA/ACh	+	+	−
Cerebral cortex	Glu	+	−	++
Cortical pyramidal layer 6	Glu	+		++
Paragigantocellular reticular nucleus	GABA/Gly	+	++	+++++
Neuroblasts, olfactory bulb	Glu	+	−	−
Striatum/Amygdala	GABA/ACh	+	+	−
Inhibitory neurons, midbrain	GABA	+	−	−
Spinal cord, Dorsal cord lamina 2-5	Glu	+	−	−
Lateral hypothalamus	Glu/TRH	+	+	−
Piriform cortex	Glu	+	+++++	−
Septal nucleus	Glu	+	−	−
Inhibitory neurons, spinal cord	GABA	+	++	−
Basket and bistratified cells, cortex/hippocampus	GABA	+	−	++
Cingulate/retrospenial area, layer 5	Glu	+	−	−
Dorsal root ganglion	Glu	+	−	−
Sleep-active interneurons, cortex/hippocampus	GABA/Sst/NO/NPY	+	−	−
Interneuron-selective interneurons, cortex/hippocampus	GABA/VIP	+	−	−
Cortical pyramidal layer 5	Glu	+	−	+
Cortical pyramidal layer 2/3	Glu	+	−	++
Piriform cortex, pyramidal neurons	Glu	+	−	−
Interneuron-selective interneurons, hippocampus	GABA/VIP	+	−	−
Nucleus of the solitary tract	Glu	+	+	−
Hippocampus CA1	Glu	+	−	−
Cingulate/retrospenial area, layer 2	Glu	+	−	−
Arcuate nucleus of the hypothalamus	GABA/AgRP/NPY	+	−	−
Inner horizontal cell, olfactory bulb	GABA	+	−	+
Superior olivary complex	GABA/Gly	+	−	−
Pons	GABA/NO	+	−	−
Ventromedial hypothalamus	Glu	+	−	−
Olfactory bulb	GABA	+	−	−
Anterior olfactory nucleus and ventral striatum	Glu	+	−	−
Superior Coliculus	GABA	+	+	−
Granule neurons, cerebellum	Glu	+	−	−
Interneuron-selective interneurons, cortex/hippocampus	GABA/VIP/CRH	+	−	−
Pallidum	GABA	+	−	−
A1-2 Noradrenergic cell groups	Glu/Nor/PrlRH	+	−	−
Superior colliculus superficial grey layer	GABA	+	+	−

Data obtained from single cell transcriptomic analysis (Zeisel et al., 2018). Expression level: - , undetected; + , very low; ++, low; +++, medium; ++++, high; +++++, very high. Ach, acetylcholine; AgRP, Agouti related peptide; CRH, corticotropin releasing hormone; Glu, Glutamate; Gly, glycine; NO, nitric oxide; Nor, norepinephrine; NPY, neuropeptide Y; PrlRH, prolactin releasing hormone; Sst, somatostatin; VIP, vasoactive intestinal peptide.

Many TRH neurons are glutamatergic (Zeisel et al., 2018). Although *Trhde* is expressed by multiple types of glutamatergic neurons, the regional distributions of *Trh* and *Trhde* mRNAs do not match (Heuer et al., 2000); in contrast, destruction of serotonin (5HT) neurons of the raphe nucleus, where TRH colocalizes with 5HT, decreases TRH content, but not TRH-DE activity, in their projections to the spinal cord (Joseph-Bravo et al., 1994), suggesting *Trhde* is not co-expressed with *Trh* in this pathway. However, the single cell transcriptomic map of the mouse brain (Zeisel et al., 2018) shows that while many *Trhde* neurons do not express *Trh*, most *Trh* cell types do express low levels of *Trhde*, while two hypothalamic *Trh* cell types are clearly positive for *Trhde*. An independent single-cell mouse transcriptomic study showed that in the medial hypothalamus there are 5 predominant clusters of *Trh* neurons, one being clearly positive for *Trhde* (Campbell et al., 2017).

Some TRH-DE neurons may be TRH-R1 or TRH-R2 positive, because the maps of the distribution of *Trhde*, *Trhr* and *Trhr2* mRNAs in the brain reveal a partial co-expression of *Trhde* and one of its receptors (Heuer et al., 2000). Data at single cell level are consistent with coexpression in some neurons, but also indicate many mismatches (Zeisel et al., 2018). An example of a site of co-expression is the medial hypothalamus, where three of the five clusters of *Trhde* neurons do express *Trhr* (Campbell et al., 2017). Furthermore, in the medial septum a large fraction of the *Trhde* cells does not express *Trhr* (Lazcano et al., 2012). We have no clues about the relationship between the subcellular localizations of TRH-DE and TRH-Rs in the cells that do express both *Trhde* and *Trhr* or *Trhr2*, in part because of the lack of TRH-DE antibodies suitable for immunocytochemistry. Existing data thus suggest no systematic association of TRH-DE with TRH receptors. What is thus the relevance of TRH-DE for TRH communication?

In brain slices, exogenous TRH is rapidly hydrolyzed, a major product being His-Pro-NH_2_, one of the two catabolites of TRH hydrolysis by TRH-DE *in vitro*, suggesting indeed that TRH-DE hydrolyzes TRH in the brain extracellular fluid (Méndez et al., 1999). In brain slices, inhibition of TRH-DE activity with CPHNA increases TRH levels recovered in the extracellular medium, with a stronger effect in regions with higher TRH-DE activity (Charli et al., 1989). Glp-Asn-Pro-Tyr-Trp-Trp-AMC also enhanced recovery of TRH released from rat brain slices (Kelly et al., 2005). Furthermore, perfusion of cortical slices with a TRH-DE inhibitor mimics the effect of TRH on the shape of action potentials generated by pyramidal neurons, suggesting that endogenous TRH released in the extracellular fluid is hydrolyzed by TRH-DE (Rodríguez-Molina et al., 2014). Thus, extracellular turnover of TRH (either exogenous or endogenous) in brain slices is relatively rapid and occurs at least in part through hydrolysis by TRH-DE.

Besides these *in vitro* studies, there is indirect evidence that TRH may be hydrolyzed through TRH-DE activity in brain *in vivo*. If TRH is hydrolyzed by TRH-DE in the brain extracellular space, the reaction product His-Pro-NH_2_ could be degraded to His-Pro by dipeptidyl peptidase IV, a widely expressed ectoenzyme (Mentlein, 1999), and/or cyclize spontaneously to His-Pro-diketopiperazine (HPDKP), which may be metabolically stable and accumulate in the brain (Prasad, 1989). HPDKP is indeed found in the brain and although diet may be an external source, part of HPDKP most likely comes from TRH hydrolysis since its concentration is reduced in the hypothalamus and cortex of TRH knockout mice (Yamada et al., 1999).

A more direct evidence for the importance of TRH-DE in controlling the effects of TRH in the brain comes from a study on the effect of TRH on ethanol-induced narcosis. One of the pharmacological effects of TRH is its capacity to enhance alertness, and reverse narcosis, and one of the brain regions that mediate this last effect is the lateral septum. An intra-medial septum injection of the TRH-DE inhibitors pGlu-Asn-Pro-AMC, or Ψ-TRH which is not an agonist of TRH-R1, enhances the effect of exogenous TRH on the duration of ethanol-induced loss of righting reflex; the time of loss of the reflex is also decreased by the injection of Ψ-TRH alone (Lazcano et al., 2012). These data give further support to the proposal that TRH-DE regulates TRH turnover *in vivo*.

Although the relevance of TRH-DE for TRH inactivation in brain requires more extensive studies, it seems reasonable to conclude that its activity may at least in some anatomical contexts be present in neuron targets of TRH or adjacent neurons, and control the turnover of TRH, and thus duration and/or intensity of TRH effect. Although the primary products of TRH-DE activity (Glp and His-Pro-NH_2_) are inactive against TRH receptors, supporting an inactivation role, the product of His-Pro-NH_2_ cyclization, HPDKP, may mimic or oppose some TRH effects, making it possible to argue that TRH-DE bio-transforms TRH (Prasad, 1995). At pharmacological doses HPDKP may be neuroprotective and improve glucose metabolism in obese and/or diabetic animals (Minelli et al., 2008). However, the physiological relevance of TRH biotransformation to HPDKP in brain is currently unknown. Thus, it seems pertinent to conclude that a major function of TRH-DE is TRH inactivation in the brain. In contrast, it remains to be determined whether Gln-Phe-Pro-NH_2_ and Gln-Tyr-Pro-NH_2_ actions are indeed terminated by TRH-DE activity *in vivo*.

## Potential Therapeutic Targeting of TRH-DE in the CNS

This section covers some of the major pharmacological properties of TRH, and of the two TRH-like peptides that are potential *in vivo* substrates of TRH-DE in the CNS, to illustrate the contexts in which TRH-DE targeting may be considered therapeutically beneficial.

Except for TRH neurons controlling the HPT axis, few experimental studies have addressed the *in vivo* physiological role of other TRH neurons. This is because of the absence of useful TRH-R antagonists *in vivo*, of the difficulty to manipulate the activity of specific TRH subtypes, and to limited information about the projections of various TRH neuron types. Some studies used strategies that correlate behavior with markers of TRH neuron activity, such as evaluation of the expression of *Trh* or cFOS, or region-specific manipulation of *Trh* expression. Along with tracing experiments, this research laid the ground to insert some TRH neurons in physiological contexts. Many studies have rather evaluated the impact of TRH or analogues on behavior and/or biochemical markers of neuronal activity. Peripheral or central (intracerebroventricular or nucleus-specific) administration of TRH or analogues produces in laboratory animals multiple behavioral changes, besides activation of the HPT axis. The mechanisms of TRH action are not fully understood, although advances have been made to characterize the electrophysiological/biochemical effects of TRH, and to identify target cells and circuits involved. Initial results led to early attempts to use TRH and analogues with improved stability, agonist potency and/or brain accessibility for experimental treatment of psychiatric or neurological diseases. A comprehensive analysis of TRH effects is beyond the scope of this review; the reader is referred to excellent reviews (Kelly, 1995; Horita, 1998; Gary et al., 2003; Daimon et al., 2013; Fröhlich and Wahl, 2019). As for other receptors, TRH or analogue action is not only limited by access and stability, but also by ligand-induced desensitization and down regulation (Sun et al., 2003; Hinkle et al., 2012). Reduced TRH receptor binding and behavioral tolerance is observed after repeated administration of TRH (Ogawa et al., 1983; Simasko and Horita, 1985), a fact that stresses one of the limits of the therapeutic use of any analogue. We will restrict the following paragraphs to some well-established effects of TRH or analogues, and whenever knowledge permits to discuss the targeting of TRH-DE.

TRH administration acutely increases arousal and locomotion; these effects are reproduced by direct injection of TRH into nucleus accumbens and septum (Horita, 1998). Other central regions are likely involved; for example, TRH increases the activity of lateral hypothalamus orexin neurons, which project to several areas involved in arousal (González et al., 2009; Hara et al., 2009). TRH-induced dopamine release has been observed in different paradigms and was proposed to be involved in TRH-induced motor activity (Kalivas et al., 1987; Yamamura et al., 1991b). The effect of TRH on arousal is also related with TRH-induced awakening from ethanol narcosis and coincides with the stimulatory effect of acute ethanol administration on *Trh* mRNA levels, accompanied with decreased *Trhde* mRNA levels and TRH-DE activity, and TRH tissue content, suggesting increased release of TRH in nucleus accumbens (de Gortari et al., 2005). In the septum, there is evidence that inhibition of TRH-DE activity promotes arousal from ethanol narcosis (Lazcano et al., 2012). Contrary to TRH, Gln-Phe-Pro-NH2 and Gln-Tyr-Pro-NH2 are not analeptic (Hinkle et al., 2002). Amplification of TRH action on arousal by TRH-DE inhibition may be therapeutically useful in niche applications.

TRH or analogue administration has generally a strong anorexic effect. The anorexic effect of TRH is probably mediated through multiple target regions; one of them is the nucleus accumbens, where TRH may interact with dopaminergic communication (Puga et al., 2016). Other regions potentially involved in the anorexic effect of TRH are various hypothalamic nuclei, such as the tuberomammillary nucleus, where TRH containing axons innervate histamine neurons (Gotoh et al., 2007; Sárvári et al., 2012), or the perifornical area/bed nucleus of stria terminalis region, from where TRH neurons may control the activity of proopiomelanocortin neurons of the arcuate nucleus (Péterfi et al., 2018). However, it was also noted that TRH injection into the brainstem promotes food intake through TRH-R1 activation (Ao et al., 2006), suggesting that control of food intake through TRH manipulation may require the optimization of the access route. There is not yet any experimental evidence showing that TRH-DE may regulate the anorexic (or orexigenic) effect of TRH.

TRH improves cognitive function (Bennett et al., 1997). TRH analogs administered to animals prevent the memory impairment caused by chronic ethanol administration or brain lesions (Bennett et al., 1997; Gary et al., 2003; Daimon et al., 2013; Meena et al., 2015). *In vivo*, TRH, or some analogs, activate the septo-hippocampal and basalis cortical cholinergic systems, eliciting acetylcholine release onto the hippocampus and cortex and restore cognitive deficits of animals with lesions in the medial septum (Itoh et al., 1994; Bennett et al., 1997; Horita, 1998; Prokai-Tatrai and Prokai, 2009; Daimon et al., 2013). The TRH analogue 2-Pyz-_L_-His (1-alkyl)-_L_-Pro-NH_2_, which exhibits high selectivity towards TRH-R2, and is more stable in rat blood plasma than TRH, possesses cognitive-enhancing activity when administered intravenously in the scopolamine-induced cognition impairment mice model, evaluated with the Morris water maze and the passive avoidance tests (Meena et al., 2015).

The hippocampus is critical in learning and memory. TRH immunoreactivity is detected in pyramidal and granular layers of CA1 and CA3, and in ventral dentate gyrus coincident with TRH binding (Hökfelt et al., 1989; Sharif et al., 1989) and with expression of *Trhr* (Calzá et al., 1992; Heuer et al., 2000). In support of the relevance of hippocampal TRH communication in learning, the status of TRH transmission is modified in the hippocampus of rats trained for 5 days in the water maze. TRH release is reduced, whereas expression of *Trh* and *Trhr* is upregulated in CA3, supporting plastic changes induced by the process of learning (Aguilar-Valles et al., 2007). TRH produces a time-dependent biphasic effect on NMDA receptor responses (Zarif et al., 2016), whereas it increases the excitability of GABA interneurons (Deng et al., 2006) in the hippocampus. Interestingly, pyramidal cells of the Ammon's horn express *Trhde* mRNA in abundance (Heuer et al., 1998b); *Trhde* may be expressed on the cell body and/or dendrites of the CA neurons. Blockade of NMDA receptors by MK801 decreases relatively rapidly TRH-DE activity in hippocampal slices, while blockade of AMPA or GABA-A receptors does not (Rodríguez-Molina et al., 2009). Additional cognitive effects of TRH may be direct onto cerebral cortex, where *Trhde* mRNA expression is also high (Heuer et al., 1998b) and TRH-DE regulates TRH action on pyramidal neurons action potential shape (Rodríguez-Molina et al., 2014). Thus, cognitive actions of TRH may be amplified by inhibition of TRH-DE activity.

Several reports show that endogenous or exogenous TRH is an anticonvulsant (Kubek et al., 1989; Sattin, 1999; Sah et al., 2011). In man, TRH is efficient for treatment of West syndrome, Lennox-Gastaut syndrome, and early infantile epileptic encephalopathy (Takeuchi et al., 2001). The electrical subthreshold stimulation produced during kindling in the amygdala or the hippocampus increases *Trh* expression or TRH content (Rosen et al., 1992; Kubek et al., 1993; de Gortari et al., 1995). Amygdala kindling increases *Trh* expression, but decreases that of *Trhr*, *Trhr2*, and *Trhde* in amygdala, and hippocampus, while only that of *Trhr2* and *Trhde* in frontal cortex (de Gortari et al., 2006). TRH content increases as kindling progresses whereas the activity of TRH-DE increases in the initial stages and decreases once kindling is established (de Gortari et al., 1995). Decreased *Trhde* expression may facilitate the protective effects of TRH. These data suggest that inhibition of TRH-DE may be protective in some forms of epilepsy.

The peripheral or central administration of TRH produces an anxiolytic effect in several animal tests (Vogel et al., 1980; Thompson and Rosen, 2000; Gutiérrez-Mariscal et al., 2008). There is evidence that endogenous TRH can perform a similar function. Thus, in animals subjected to the defensive burying test, there is a specific inhibition of TRH release and *Trh* mRNA levels in amygdala but not in other limbic areas (Gutiérrez-Mariscal et al., 2008). Furthermore, amygdalar *Trh* expression and anxiety behavior are inversely correlated in the elevated plus maze and in the open field test (Gutiérrez-Mariscal et al., 2012). In adult male rats, exposure to an open field test during the inactive phase increases *Trh* expression in the cortical nucleus of the amygdala, an area involved in processing fear stimuli (Gutiérrez-Mariscal et al., 2008). *Trhr* KO mice are more anxious than wild type relatives (Zeng et al., 2007) while those deficient in *Trhr2* exhibit reduced anxiety (Sun et al., 2009). It is warranted to test the status of *Trhde* KO mice.

Experimental evidence for an antidepressant effect of TRH in animal models and humans is controversial, although *Trhr2*-deficient mice are euthyroid and exhibit a depressive behavior (Sun et al., 2009). As an example of controversy, the injection of TRH into rats decreases the immobility time in the Porsolt test (Drago et al., 1990) whereas the injection of Taltirelin into the amygdala increases the immobility time in chronic-stress-induced depressed mice. In the depressed mice, *Trh* and *Trhr* expression in the basolateral amygdala is increased after 14 days of 2 h of daily restraint (Choi et al., 2015). This result seems to contradict the reported anxiolytic effect of TRH. Species and strain differences in stress responses and the previous stress history of the animal (Joseph-Bravo et al., 2015a) may explain some of these discrepant results. Finally, Gln-Tyr-Pro-NH_2_ is active in the Porsolt Swim Test (Pekary et al., 2005). Since mood stabilizing valproate acutely enhances, among other TRH-like peptides, Gln-Tyr-Pro-NH_2_ concentration in brain regions and most notably in pyriform cortex (Pekary et al., 2004), it will be relevant to take Gln-Tyr-Pro-NH_2_ into account if TRH-DE is targeted for an anti-depressant effect.

Several neurologic disorders improve with treatment with TRH and some of its stable analogs. This has been shown for scopolamine-induced memory loss, prolonged impaired consciousness (Horita, 1998; Meena et al., 2015), and neuronal death occurring after a transient ischemic attack (TIA) (Shishido et al., 1999). In rats, *Trh* expression increases in peri-ischemic tissue, while that of *Trhde* drops drastically. The response is independent of age of induction of TIA, and post-ischemia time. This may be a TRH protective effect, amplified by *Trhde* down regulation (Buga et al., 2012). Neuroprotective actions of TRH analogs include protection against neuronal injury induced by excitotoxicity and oxidative stress, and inflammation (Gary et al., 2003; Daimon et al., 2013; Fröhlich and Wahl, 2019). In the spinal cord, TRH may be involved in motor activity control and pain modulation; its administration promotes the recovery from acute spinal cord injury and has been used clinically with promising results (Fehlings and Baptiste, 2005). Taltirelin, which is degradation resistant, has been accepted in the treatment of spinocerebellar degeneration (SCD) (Kinoshita et al., 1994; Kinoshita et al., 1998). Taltirelin has anti-ataxic and neuroprotective actions (Urayama et al., 2002; Nakamura et al., 2005; Veronesi et al., 2007). Finally, JAK4D elicits large neuroprotective effects in neurodegenerative animal models. Systemic administration of JAK4D reduces cognitive deficits in a kainate (KA)-induced rat model of neurodegeneration, protects against free radical release and neuronal damage evoked by KA in rat, and reduces motor decline and lumbar spinal cord neuronal loss in a transgenic Amyotrophic Lateral Sclerosis mice model (Kelly et al., 2015). These data point to the importance of targeting TRH-DE to improve treatment of neurodegenerative diseases with TRH derivatives.

TRH analogs resistant to degradation by TRH-DE are promising for treating some neurological disorders. However, autonomic effects of these analogs need to be considered. Central TRH application has effects on the respiratory, cardiac and gastric systems. Most of these actions are due to targets of the TRH neurons present in brain stem. TRH is synthesized in raphé pallidus, obscurus, and para-pyramidal neurons that project to sympathetic preganglionic neurons of the intermediolateral (IML) cell column of the spinal cord and to vagal motor neurons located in the dorsal vagal complex (Taché et al., 2006). Some of TRH neurons that project to the IML use serotonin and substance P as co-transmitters; depending on the complement of receptors in target neurons, TRH participates in several aspects of respiratory network control (Hodges and Richerson, 2008).

In the vagal complex TRH activates neurons of the dorsal motor nucleus of the vagus that project to visceral organs such as pancreas regulating insulin secretion (Yang et al., 2002), or as stomach, stimulating gastric secretion (Tache, 2012). TRH-TRH-R1 signaling in brain stem, or TRH administered in low doses, have protective action against damaging agents in the gastric mucosa, whereas this is not the case in high doses as injections into the cisterna magna induce a dose-related activation of gastric vagal efferent discharges in rats. TRH applied in the hindbrain modulates gastric secretion and motility, blood pressure, hepatic blood flow, stimulates brown adipose tissue thermogenesis and respiratory output (Taché et al., 2006; Hou et al., 2012; Yang et al., 2012; Pilowsky, 2014). Although TRH-DE activity is lower in the brain stem than in other regions of the brain (Vargas et al., 1992a), the potential to alter autonomic functions if TRH-DE is affected cannot be underestimated.

In conclusion, at CNS level, many TRH effects, and some Gln-Phe-Pro-NH2 and Gln-Tyr-Pro-NH2 actions, may have a therapeutic potential (i.e. antiepilepsy, anxiolysis, improvement of learning, neuroprotection). The presence of TRH-DE in many of the sites of action of TRH suggests that inhibition of TRH-DE activity should increase the action of endogenous TRH. This may produce results that are better suited than those sought with TRH analogues.

## TRH-DE and the Thyroid Axis

This topic has been recently reviewed (Rodríguez-Rodríguez et al., 2019) and will only be briefly discussed here. Although a large part of CNS *Trhde* is expressed in neurons, a few years ago it was discovered that *Trhde* is expressed in tanycytes of the hypothalamus, a glial cell type localized at the base and ventrolateral walls of the third ventricle of the hypothalamus (Sánchez et al., 2009). All sub-types of tanycytes express *Trhde*; the β2 tanycytes, which send cytoplasmic extensions into the external layer of the median eminence where they are close to TRH terminals of the parvocellular TRHergic neurons of the PVN, in proximity of the portal capillaries that transport TRH to the anterior pituitary have the highest expression of *Trhde* (Sánchez et al., 2009). In median eminence explants, TRH-DE inhibition enhances TRH recovered from the incubation medium, suggesting that the enzyme actively hydrolyses TRH in the median eminence extracellular space (Sánchez et al., 2009). Thus, after release TRH may be partially hydrolyzed by TRH-DE before entry into the portal vessels. A physiological consequence would be that the amount of TRH reaching the anterior pituitary would not only depend on the activity of the TRH neurons, but also on median eminence TRH-DE activity, which would regulate the intensity of TRH-induced TSH secretion according to clues detected at median eminence level. Consistent with this idea, it was shown that although the intraperitoneal injection of HcPI in control animals does not change serum concentration of TSH, it does increase it in cold stressed animals (Sánchez et al., 2009). Although HcPI inhibits TRH-DE activity inside the blood–brain barrier, as well in the anterior pituitary and serum, *Trhde* expression is not detected in thyrotropes, and inhibition of TRH-DE expression or activity does not enhance TRH-induced TSH release (Bauer et al., 1990; Cruz et al., 2008); this evidence strongly suggests that anterior pituitary TRH-DE is not critical for the control of TSH release. Finally, the role of the serum enzyme, that is produced by the liver (Schmitmeier et al., 2002) cannot be discarded, since this isoform likely circulates through the portal vessels. Although a definitive demonstration for control of TRH entry into the portal capillaries by tanycyte TRH-DE is required, evidence about regulation of TRH-DE activity is also consistent with this proposal.

A major mode of control of the activity of the HPT axis is negative feedback at various levels, including inhibition of *Trh* mRNA synthesis in the hypophysiotropic neurons of the PVN (Fekete and Lechan, 2014). At the level of the tanycytes, *Trhde* expression and activity are rapidly (in a few hours) stimulated by peripheral injection of T4, thus putatively contributing to the negative feedback (Sánchez et al., 2009). The effect of T4 on *Trhde* depends on the local conversion of T4 to T3 by deiodinase 2 *(Dio2*) also expressed in tanycytes (Marsili et al., 2011). The mechanism by which TH induces *Trhde* expression is not known; thyroid hormone response elements have not been detected in the vicinity of the putative transcription initiation of *Trhde* (**Figure 1**), although many genes regulated by TH do not contain them (Chatonnet et al., 2013).

The HPT axis is regulated by energy balance (Fekete and Lechan, 2014; Joseph-Bravo et al., 2015b). Apart from down regulation of TRH neurons activity, a prolonged fast enhances *Dio2* and *Trhde* expression and activity in the male rat median eminence (Diano et al., 1998; Lazcano et al., 2015). The transient induction of *Trhde* expression may contribute to prolong the downward adjustment of the HPT axis, along with an increase in thyroliberinase activity, in spite of a partial reactivation of *Trh* synthesis by the PVN at 72 h fasting (Lazcano et al., 2015). Other data show that diet-induced obesity (with unsaturated fat) enhances median eminence *Trhde* expression in male rats (Jaimes-Hoy et al., 2019), suggesting that the relationship between energy balance and activity of TRH-DE is complex. Finally, *Trhde* expression in the median eminence is programed by neonatal stress, along with other aspects of HPT axis function (Jaimes-Hoy et al., 2016). It is thus evident that long-term events shape median eminence TRH-DE activity.

In these models, there are few clues about the immediate regulators of *Trhde* expression/activity, and it has not been shown that *Trhde* adjustments are critical for HPT axis activity. The local thyroid status is probably involved (Lazcano et al., 2015) and besides hormonal signals, tanycytes are responsive to neuronal inputs. Recently it was shown that the release of TRH in the median eminence activates the TRH-R1 receptor on the surface of tanycytes, resulting in two rapid events: an increase of the surface of tanycyte end-feets covering the portal capillaries, and an increase in TRH-DE activity in the median eminence, both of which may reduce TRH access to the capillary bed. Furthermore, a long term (days) blockade of Gq transduction decreased TRH-DE activity. Thus, the firing of TRH neurons likely regulates TRH-DE activity and localization. This coordinated mechanism may help to shape the pulses of TRH that reach the anterior pituitary (Müller-Fielitz et al., 2017).

An intriguing question is the relevance of TRH-like peptides sensitive to hydrolysis by TRH-DE in the control of the HPT axis. Gln-Phe-Pro-NH_2_ is released from hypothalamic slices (Méndez et al., 1999), Gln-Phe-Pro-NH_2,_ as well as TRH, is detected in rat thyroid (Rausell et al., 1999; Smyth et al., 1999) and Gln-Phe-Pro-NH_2_ enhances serum T3 concentration (Cremades et al., 1998). Thus, median eminence and thyroid gland may be sites where the functional relationship between Gln-Phe-Pro-NH_2_ and TRH-DE warrants further investigation.

## Targeting Median Eminence TRH-DE to Control Thyroid Status?

Thyroid diseases are very common and a major one is sub-clinical or clinical hypothyroidism, which is mainly treated with T4 (and T3). Although this therapeutic option is successful, it is not perfect and there is room for improvement. Among the major problems of the therapeutic use of TH is the risk of producing unattended hyperthyroidism and thus cardiovascular or skeletal consequences. These problems have led to significant avenues of research, to limit unintended effects. One of these approaches has been the development of specific agonists of TH receptor β (Runfola et al., 2020). Another focus has been on the development of thyroid hormone-peptide complexes, to direct TH to the tissues that express the peptide receptor (Finan et al., 2016).

Peripheral inhibition of peptidase activity has been quite successful in various contexts (hypertension, type 2 diabetes). Although requiring confirmatory experiments, the weight of evidence strongly suggests that in rats median eminence TRH-DE activity controls the output of TRH and thus TSH secretion, at least during bouts of TRH neurons activity. This suggests that inhibition of this enzyme may transiently increase TRH-induced TSH secretion, enhancing the hypothalamic signal that promotes TSH secretion in conditions of insufficient drive. Because β2 tanycyte TRH-DE activity is outside the blood brain barrier, it should be possible to target this compartment and enhance the extracellular concentration of TRH in the portal vessels, without affecting TRH communication in the rest of the CNS. Furthermore, the narrow specificity of TRH-DE should facilitate the development of agents that have no impact on other peptides turnover. The final purpose of this manipulation would be to enhance thyroid hormone secretion without changing the natural pattern of control of the HPT axis and to produce self-regulated and small effects on TH concentration, reducing cardiovascular and bone effects.

Various milestones should be reached to target peripheral TRH-DE activity to improve thyroid status in hypothyroidism. One is to characterize the long-term impact of TRH-DE inhibition on HPT axis parameters, since the axis can self-regulate efficiently; for example, the long-term ablation of tanycytes does not change serum TSH concentration (Yoo et al., 2019). Another consideration is that changes in serum TSH concentration may have non thyroidal effects. Likewise, prolactin secretion would have to be monitored, to exclude alteration of this critical hormone. Finally, the distribution and role of TRH-DE activity in the periphery requires clarification.

## TRH-DE and Prolactin Secretion

In mammals, apart from the negative control of prolactin secretion by hypothalamic tuberoinfundibular dopamine neurons, hypothalamic prolactin releasing factors are also important (Grattan, 2015). TRH is not only a thyrotropin releasing factor but also a prolactin releasing factor. TRH is necessary to maintain maximal prolactin output in lactating mice (Yamada et al., 2006). In primary cultures of female rat anterior pituitary cells, *Trhde* expression is detected in some lactotropes, and inhibition of TRH-DE expression or activity enhances TRH-induced prolactin secretion (Bauer et al., 1990; Cruz et al., 2008). If the importance of *Trhde* for the control of prolactin secretion, which is contingent on many factors, is confirmed in relevant *in vivo* models, inhibition of anterior pituitary TRH-DE activity might increase prolactin secretion.

## TRH-DE in Peripheral Tissues

**Figure 3:**
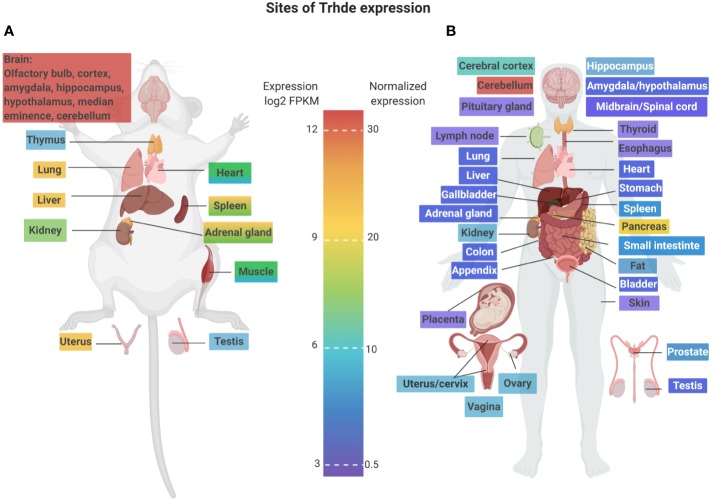
Potential sites of targeting of TRH-DE in rats and humans. *Trhde* is mainly expressed in a few major organs; in rats, gene expression is higher in brain followed by lung, liver and uterus; in humans, major sites of expression are cerebellum and pancreas. Panel **(A)** illustrates tissue *Trhde* expression distribution in rat (log2 FPKM, fragments per kilobase of transcript per million mapped reads) based on the database of the rat body map available from http://pgx.fudan.edu.cn/ratbodymap/index.html (Yu et al., 2014); some of the brain regions which express medium (i.e. hypothalamus) to high mRNA levels are also indicated (Heuer et al., 1998b). Panel **(B)** illustrates tissue *Trhde* expression distribution in humans based on the Human Protein Atlas available from http://www.proteinatlas.org (Uhlén et al., 2015). Colors on organ/tissue names represent expression levels of *Trhde*. Information regarding sex and age of the donor, can be found at http://www.proteinatlas.org/about/cellines. Figure was created with Biorender.com.

Although targeting of CNS and/or median eminence/pituitary TRH-DE activity has experimental support, a much less understood aspect is the function of *Trhde* expressed in the periphery. This must be fully assessed for establishing the safety profile of the peripheral inhibition of TRH-DE activity or expression. **Figures 2** and **3** illustrate that although the brain is a major source of *Trhde* expression, developmentally regulated expression of *Trhde* is also detected in multiple rat tissues, of both sexes. However, it should be noted that in rats and rabbits, the peripheral tissues have low levels of TRH-DE activity (Vargas et al., 1992a). Human transcriptomic data indicate baseline expression of *Trhde* in various peripheral organs, being more abundant in kidney, spleen, ovaries, uterus, adipose tissue, small intestine Peyer's patch, duodenum, pancreas, and lung, and lower in colon, prostate, stomach and testis. In these tissues, *Trhde* expression is detected in adults. For kidney, lung, stomach and testis, *Trhde* expression has also been reported at early stages of embryonic development (“ensembl.org: TRHDE—ENSG00000072657,” n.d. and “bgee.org: TRH-DE—ENSG00000072657”) (**Figure 3**). We review below the state of knowledge about TRH-DE in the best-studied tissues.

Thyroliberinase is a liver specific isoform of TRH-DE (Schmitmeier et al., 2002) that is secreted into the general circulation. It is the only enzyme that degrades TRH in rat serum (Friedman and Wilk, 1985). Its role is puzzling; it probably reaches the hypothalamic-pituitary portal vessels, but its specific relevance for anterior pituitary control is untested. Thyroliberinase may also reduce the buildup of TRH concentration in the peripheral tissues where TRH may act as a paracrine effector (Morley, 1979). Finally, it should contribute significantly to the limited efficiency of the parenteral injection of TRH, being an interesting target to enhance the efficacy of parenteral TRH analogues.

One of the peripheral tissues in which TRH presence was noticed early is the rat pancreas, where it is concentrated in the islets of Langerhans (Martino et al., 1978). A transient prenatal expression of *Trh* is detected in pancreatic β-cells, which coincides with development of insulin secretory activity (Yamaoka and Itakura, 1999; Basmaciogullari et al., 2000; Štrbák, 2018). In rodents, *Trhr* is expressed in the pancreas, including in β-cells (Yamada et al., 2000; Luo and Yano, 2004); a potential effect of TRH-TRH-R1 is the promotion of programmed cell death during development (Mulla et al., 2009). *Trh* KO mice have an increased glycemia, which may be the result of reduced secretion of insulin in response to high glucose concentration (Yamada et al., 1997). On the other hand, *Trhr* KO mice are hypothyroid and hyperglycemic (Zeng et al., 2007), while *Trhr2* KO mice are euthyroid and normoglycemic (Sun et al., 2009). Gln-Phe-Pro-NH2 is detected in pancreas, where it attenuates TRH effect on insulin secretion in perifused islets (Kulkarni et al., 1995). Although pancreatic *Trhde* expression is significant in human (**Figure 3**), there is no evidence for TRH-DE activity in developing or adult rat (Vargas et al., 1992b). *Trhde* KO mice are normoglycemic (Tang et al., 2010). The relevance of TRH-DE in human pancreatic function requires an urgent investigation; is expression leading to TRH-DE activity, or to non-active isoforms?

*Trh*, and *Trhr* are expressed in the heart, in fibroblasts and in cardiomyocytes (Carnell et al., 1992; Shi et al., 1996); TRH, as well as Gln-Phe-Pro-NH_2_ and Gln-Tyr-Pro-NH_2_, are detected in heart tissue, where values vary along the circadian cycle (Pekary and Sattin, 2017). Various evidences suggest that heart TRH might be relevant in cardiovascular disease. TRH induces hypertrophic and fibrotic markers in heart cells (Schuman et al., 2014). Overexpression of *Trh* in left ventricular cells promotes heart hypertrophy in rats (Jin et al., 2004). Spontaneously hypertensive rats overexpress *Trh* in the left ventricle, and reduction of *Trh* expression inhibits fibrosis and cardiomyocyte enlargement (Schuman et al., 2011). Furthermore, angiotensin II–induced heart hypertrophy depends on *Trh* expression (Peres Diaz et al., 2018). Thus, TRH may have a substantial role in cardiac hypertrophy. *Trhde* is expressed in the heart of Fischer-344 male and female rats, with noticeable developmental increases (**Figure 2**) (Yu et al., 2014). In the human heart, *Trhde* transcripts can be detected at 10 weeks (Szabo et al., 2015). It will be important to clarify whether TRH-DE activity is present in heart and controls local TRH and/or Gln-Phe-Pro-NH_2_ and Gln-Tyr-Pro-NH_2_ actions.

TRH-like immunoreactivity is detected in various organs of the digestive system (Leppäluoto et al., 1978). TRH levels are high in gastric juice and mucosa (Nishio et al., 1999). In the digestive system, while TRH-R1 presence in the stomach is scarce (Nishio et al., 1990), TRH-R1 has been detected by immunohistochemistry in the small intestine (Mitsuma et al., 1995), where TRH excites submucus neurons and enhances heat shock protein 60 expression (Zafirov et al., 1991; Sasahara et al., 1998). One subtype of cholinergic neuron, in the myenteric plexus of the mouse small intestine, is characterized by a very high expression of *Trhde* (Zeisel et al., 2018) (**Table 1**), but these neurons, which may receive vagal afferents, do not express *Trhr* or *Trhr2*. In this system, TRH-DE may inactivate TRH produced and released locally. However, as for the heart, there is yet no evidence that TRH-DE activity is present in the intestinal mucosa. Because of the high relative expression of *Trhde* in neurons of the enteric system, potential adverse effects of TRH-DE manipulation should be considered. In humans, intestinal *Trhde* expression initiates around 10–20 weeks of age (Szabo et al., 2015), being significant in adults. An accumulation of neuropeptides, including TRH, in the lumen of the colon of patients with inflammatory bowel disease (Yamamoto et al., 1996) is noticeable; animal models of the disease might shed light about TRH-DE role in this pathology, if any.

TRH has significant effects over the function of immune system, some of which dependent on pituitary control (Kamath et al., 2009). Hypophysiotropic TRH neurons are involved in inflammation responses dependent and independent of T-cell activation. LPS administration doesn't induce T-cell activation and is linked with an initial reduction of *Trh* expression in the PVN (Kamath et al., 2009). However, T-cell dependent inflammation induces the opposite, with the response dependent on hypophysiotropic TRH neurons and prolactin secretion from the pituitary (Perez Castro et al., 1999). Although the site of TRH production acting directly on immune cells is unknown, TRH-R1 can be detected in lymphoid tissues, including the thymus (Montagne et al., 1999) and bone marrow (Matre et al., 2003). Administration of TRH in rats increases the proliferation of cells of the thymus (Pawlikowski et al., 1992) and protects against its involution and reduction of the count of lymphocytes in peripheral blood, induced by lateral hypothalamus lesion (Lesnikov et al., 1992). TRH may thus be of therapeutic value in diseases dependent on immune system activation. Administration of TRH protects against pathogens such as encephalomyelitis virus (Pierpaoli and Yi, 1990), and candidiasis (Blaszkowska et al., 2004). Transcriptomic analysis reveals that *Trhde* is expressed in thymus, with a significant drop of expression in aging rats (**Figure 2**) (Yu et al., 2014). Although TRH-DE activity and function have not been determined in lymphoid organs, TRH-DE inhibitors could be interesting alternatives to TRH treatment in inflammatory diseases.

In the reproductive system, Leydig cells express both *Trh* and *Trhr* (Pekary and Sattin, 2017). Repeated administration of TRH counters aging-induced alteration of rat testicular structure and function (Pierpaoli, 2013). Gln-Phe-Pro-NH_2_ is also expressed by the prostate and detected in seminal fluid (Bilek et al., 1992; Khan et al., 1992; Gkonos et al., 1994); however, its local effect is unknown. In females, TRH increases the contractility of urethral and vaginal muscle (Zacur et al., 1985) and Gln-Phe-Pro-NH2 is detected in the mammary gland (Ghilchik et al., 2000). Since low levels of expression of *Trhde* occur in various reproductive organs (**Figure 3**), its relevance for TRH or Gln-Phe-Pro-NH_2_ actions may not be important but should nevertheless be investigated.

In conclusion, there is little evidence that, apart from pituitary and liver, other peripheral tissues which express *Trhde* give rise to significant TRH-DE activity. It remains possible that in some tissues expression might be mainly attributed to the mRNA coding for the truncated version of TRH-DE (Chavez-Gutierrez et al., 2005) and that TRH-DE* is not only a dominant negative isoform of TRH-DE but is also a non-catalytic protein. However, *Trhde* KO mice do not show any obvious problem in standard conditions, except for a small decrease of body weight (Tang et al., 2010). Finally, it will be critical to clarify the extent of TRH-DE activity in human tissues.

## Conclusions and Challenges

With the available information of the wide distribution of TRH receptors, and the multiple functions TRH displays, the pharmacological use of agonists of the TRH-R1 receptors that resist degradation by TRH-DE might impact on many functions.

Albeit alternatives are not completely excluded yet, it appears that hydrolysis by the TRH degrading ectoenzyme in the serum and the extracellular space of the brain, including the median eminence, is a critical pathway for TRH inactivation. The relevance of this enzyme for Gln-Phe-Pro-NH_2_ and Gln-Tyr-Pro-NH_2_ inactivation remains to be investigated. Since most central pharmacological effects of peptidase resistant analogues of TRH are beneficial in rodents, and there is some consistent evidence in humans, targeting central TRH-DE with specific inhibitors may have significant advantages over TRH agonists in some central diseases, in particular to produce more focused effects according to the natural activity of the TRH neurons. On the other hand, the limited span of the distribution of TRH-DE activity in the periphery opens the possibility that inhibition of median eminence TRH-DE activity may be an interesting target for treatment of hypothyroidism and may have few non-intended targets, if a compound that does not pass the blood-brain barrier is chosen. However, it will be necessary to understand the full extent of the distribution of peripheral TRH-DE activity and function in humans before this idea proceeds further.

Targeting the inactivation process in the brain will increase ligand concentration in the synaptic space and increase TRH effect, alike the mechanism of action of drugs that inhibit neurotransmitter uptake. However, an intrinsic problem lies on the stringent specificity of TRH-DE for the TRH structure. Thus, it is not surprising that some of the TRH-DE inhibitors function also as TRH-R agonists (Scalabrino et al., 2007) and may thus promote receptor down regulation or, undesirable side effects. For example, even for a more stable analog of TRH as Taltirelin, hyperprolactinemia is induced (Kanasaki et al., 2011). Even if the analog does not produce endocrine effects in humans, other peripheral effects such as induction of gastric erosion and hypermotility of the intestine may occur. The challenge to design a specific inhibitor to TRH-DE not recognized by TRH-R1 has been overcome with Ψ-TRH design, although better chemistries and affinities are required. In theory, this should be possible, as has been done for other proteases (Patchett and Cordes, 1985; Traube et al., 2014; Lai et al., 2015).

Even if better inhibitors are found, unknowns remain. Are Gln-Phe-Pro-NH_2_ and Gln-Tyr-Pro-NH_2_ physiologically relevant substrates of TRH-DE? Does inhibition of TRH-DE activity has concrete effects in preclinical models? These, and other milestones, need to be passed to determine if manipulation of TRH-DE expression or activity has therapeutic potential.

## Author Contributions

J-LC conceived and wrote the manuscript. PJ-B conceived and wrote the manuscript. AR-R generated figures and table and edited the manuscript. KH-O, AC-V, RU, and LJ-H wrote specific parts of the manuscript.

## Funding

Supported by grants from DGAPA-UNAM to J-LC (PAPITT IN206712), and AC-V (PAPITT IN 206416 and 212719), and from CONACYT to JLC (PN562; CB254960).

## Conflict of Interest

The authors declare that the research was conducted in the absence of any commercial or financial relationships that could be construed as a potential conflict of interest.

## References

[B1] Aebi M. (2013). N-linked protein glycosylation in the ER. Biochim. Biophys. Acta (BBA) Mol. Cell Res. 1833, 2430–2437. 10.1016/j.bbamcr.2013.04.001 23583305

[B2] Aguilar-Valles A. Sánchez E. de Gortari P. García-Vazquez A. I. Ramírez-Amaya V. Bermúdez-Rattoni F. (2007). The expression of TRH, its receptors and degrading enzyme is differentially modulated in the rat limbic system during training in the Morris water maze. Neurochem. Int. 50, 404–417. 10.1016/j.neuint.2006.09.009 17101195

[B3] Amenta F. Sabbatini M. Coppi G. Maggioni A. Olgiati V. Panocka I. (1997). Effect of treatment with the neuroactive peptide posatirelin on microanatomical changes of frontal cortex and hippocampus caused by lesions of the locus coeruleus. Drugs Exp. Clin. Res. 23, 77–88. 9309383

[B4] Ao Y. Go V. L. W. Toy N. Li T. Wang Y. Song M. K. (2006). Brainstem Thyrotropin-Releasing Hormone regulates food intake through vagal-dependent cholinergic stimulation of ghrelin Secretion. Endocrinology 147, 6004–6010. 10.1210/en.2006-0820 16959836

[B5] Ashworth R. Yu R. Nelson E. J. Dermer S. Gershengorn M. C. Hinkle P. M. (1995). Visualization of the thyrotropin-releasing hormone receptor and its ligand during endocytosis and recycling. Proc. Natl. Acad. Sci. 92, 512–516. 10.1073/pnas.92.2.512 7831321 PMC42771

[B6] Awadé A. C. Cleuziat P. H. GonzalèS T. H. Robert-Baudouy J. (1994). Pyrrolidone carboxyl peptidase (Pcp): An enzyme that removes pyroglutamic acid (pGlu) from pGlu-peptides and pGlu-proteins. Proteins 20, 34–51. 10.1002/prot.340200106 7824521

[B7] Bagul P. Khomane K. S. Kesharwani S. S. Pragyan P. Nandekar P. P. Meena C. L. (2014). Intestinal transport of TRH analogs through PepT1: the role of *in silico* and *in vitro* modeling: role of structural modifications on PepT1-mediated transport. J. Mol. Recognit. 27, 609–617. 10.1002/jmr.2385 25178856

[B8] Basmaciogullari A. Cras-Meneur C. Czernichow P. Scharfmann R. (2000). Pancreatic pattern of expression of thyrotropin-releasing hormone during rat embryonic development. J. Endocrinol. 166, 481–488. 10.1677/joe.0.1660481 10974642

[B9] Bauer K. Nowak P. Kleinkauf H. (1981). Specificity of a serum peptidase hydrolyzing thyroliberin at the pyroglutamyl-histidine bond. Eur. J. Biochem. 118, 173–176. 10.1111/j.1432-1033.1981.tb05501.x 6116600

[B10] Bauer K. Carmeliet P. Schulz M. Baes M. Denef C. (1990). Regulation and cellular localization of the membrane bound Thyrotropin-Releasing Hormone-degrading enzyme in primary cultures of neuronal, glial and adenohypophyseal cells. Endocrinology 127, 1224–1233. 10.1210/endo-127-3-1224 2117525

[B11] Bauer K. Heuer H. Ifflander F. Peters A. Schmitmeier S. Shomburg L. (1997). ““Inactivation of thyrotropin-releasing hormone (TRH) by a TRH-specific ectoenzyme,”,” in Cell-Surface Peptidases in Health and Disease. Eds. Kenny B. AJ C. M. (UK: Oxford: BIOS Scientific Publishers), 239–248.

[B12] Bauer K. (1979). Thyroliberin analogues as competitive inhibitors of thyroliberin degradation by brain enzymes. Hoppe-Seyler's Z Physiol. Chem. 360, 1126.

[B13] Bauer K. (1994). Purification and Characterization of the Thyrotropin-releasing-hormone-degrading Ectoenzyme. Eur. J. Biochem. 224, 387–396. 10.1111/j.1432-1033.1994.00387.x 7925352

[B14] Bellemere G. Vaudry H. Morain P. Jegou S. (2005). Effect of prolyl endopeptidase inhibition on arginine-vasopressin and Thyrotrophin-Releasing Hormone catabolism in the rat brain. J. Neuroendocrinol. 17, 306–313. 10.1111/j.1365-2826.2005.01308.x 15869566

[B15] Bennett G. W. Ballard T. M. Watson C. D. Fone K. C. F. (1997). Effect of neuropeptides on cognitive function. Exp. Gerontol. 32, 451–469. 10.1016/S0531-5565(96)00159-3 9315449

[B16] Bidaud I. Galas L. Bulant M. Jenks B. G. Ouwens D. T. W. M. Jégou S. (2004). Distribution of the mRNAs encoding the thyrotropin-releasing hormone (TRH) precursor and three TRH receptors in the brain and pituitary of *Xenopus laevis*: Effect of background color adaptation on TRH and TRH receptor gene expression: proTRH and *x* TRHR mRNAs in Brain and Pituitary of *Xenopus*. J. Comp. Neurol. 477, 11–28. 10.1002/cne.20235 15281077

[B17] Bilek R. Gkonos P. J. Tavianini M. A. Smyth D. G. Roos B. A. (1992). The thyrotrophin-releasing hormone (TRH)-like peptides in rat prostate are not formed by expression of the TRH gene but are suppressed by thyroid hormone. J. Endocrinol. 132, 177–184. 10.1677/joe.0.1320177 1541917

[B18] Blaszkowska J. Pawlikowski M. Komorowski J. Kurnatowski P. (2004). Effect of thyroliberin on the course of experimental candidosis in mice. Thyroliberin-Wirkung auf den Verlauf der experimentellen Candidose der Maus. Mycoses 47, 115–120. 10.1111/j.1439-0507.2004.00961.x 15078427

[B19] Buga A.-M. Scholz C. J. Kumar S. Herndon J. G. Alexandru D. Cojocaru G. R. (2012). Identification of new therapeutic targets by genome-wide analysis of gene expression in the ipsilateral cortex of aged rats after stroke. PloS One 7, e50985. 10.1371/journal.pone.0050985 23251410 PMC3521001

[B20] Bundgaard H. Møss J. (1990). Effect of thyroliberin on the course of experimental candidosis in mice. Pharmaceut. Res. 07, 885–892. 10.1023/A:1015933504191

[B21] Calzá L. Giardino L. Ceccatelli S. Zanni M. Elde R. Hökfelt T. (1992). Distribution of thyrotropin-releasing hormone receptor messenger RNA in the rat brain: An in situ hybridization study. Neuroscience 51, 891–909. 10.1016/0306-4522(92)90528-A 1488129

[B22] Campbell J. N. Macosko E. Z. Fenselau H. Pers T. H. Lyubetskaya A. Tenen D. (2017). A molecular census of arcuate hypothalamus and median eminence cell types. Nat. Neurosci. 20, 484–496. 10.1038/nn.4495 28166221 PMC5323293

[B23] Carnell N. E. Feng P. Kim U. J. Wilber J. F. (1992). Preprothyrotropin-releasing hormone mRNA and TRH are present in the rat heart. Neuropeptides 22, 209–212. 10.1016/0143-4179(92)90047-Z 1508323

[B24] Chávez-Gutiérrez L. Matta-Camacho E. Osuna J. Horjales E. Joseph-Bravo P. Maigret B. (2006). Homology modeling and site-directed mutagenesis of pyroglutamyl peptidase II. Insights into omega-versus aminopeptidase specificity in the M1 family. J. Biol. Chem. 281, 18581–18590. 10.1074/jbc.M601392200 16611635

[B25] Charli J.-L. Ponce G. McKelvy J. F. Joseph-Bravo P. (1984). Accumulation of Thyrotropin Releasing Hormone by rat hypothalamic slices. J. Neurochem. 42, 981–986. 10.1111/j.1471-4159.1984.tb12700.x 6422001

[B26] Charli J. L. Mendez M. Joseph-Bravo P. Wilk S. (1987). Specific inhibitors of pyroglutamyl peptidase I and prolyl endopeptidase do not change the in vitro release of TRH or its content in rodent brain. Neuropeptides 9, 373–378. 10.1016/0143-4179(87)90010-2 2886950

[B27] Charli J.-L. Cruz C. Vargas M.-A. Joseph-Bravo P. (1988). The narrow specificity pyroglutamate amino peptidase degrading TRH in rat brain is an ectoenzyme. Neurochem. Int. 13, 237–242. 10.1016/0197-0186(88)90060-5 20501293

[B28] Charli J.-L. Mendez M. Vargas M.-A. Cisneros M. Assai M. Joseph-Bravo P. (1989). Pyroglutamyl peptidase II inhibition specifically increases recovery of TRH released from rat brain slices. Neuropeptides 14, 191–196. 10.1016/0143-4179(89)90044-9 2575716

[B29] Charli J. L. Vargas M. A. Cisneros M. de Gortari P. Baeza M. A. Jasso P. (1998). TRH inactivation in the extracellular compartment: role of pyroglutamyl peptidase II. Neurobiol. (Bp) 6, 45–57. 9713831

[B30] Chatonnet F. Guyot R. Benoit G. Flamant F. (2013). Genome-wide analysis of thyroid hormone receptors shared and specific functions in neural cells. Proc. Natl. Acad. Sci. 110, E766–E775. 10.1073/pnas.1210626110 23382204 PMC3581916

[B31] Chavez-Gutierrez L. Bourdais J. Aranda G. Vargas M. A. Matta-Camacho E. Ducancel F. (2005). A truncated isoform of pyroglutamyl aminopeptidase II produced by exon extension has dominant-negative activity. J. Neurochem. 92, 807–817. 10.1111/j.1471-4159.2004.02916.x 15686482

[B32] Choi J. Kim J. Kim T.-K. Park J.-Y. Lee J.-E. Kim H. (2015). TRH and TRH receptor system in the basolateral amygdala mediate stress-induced depression-like behaviors. Neuropharmacology 97, 346–356. 10.1016/j.neuropharm.2015.03.030 26107116

[B33] Colson A. Gershengorn M. (2006). Thyrotropin-Releasing Hormone analogs. MRMC 6, 221–226. 10.2174/138955706775476019 16472189

[B34] Cornford E. M. Braun L. D. Crane P. D. Oldendorf W. H. (1978). Blood-brain barrier restriction of peptides and the low uptake of enkephalins. Endocrinology 103, 1297–1303. 10.1210/endo-103-4-1297 744146

[B35] Cremades A. Peñafiel R. Rausell V. Del Rio-Garcia J. Smyth D. G. (1998). The thyrotropin-releasing hormone-like peptides pGlu-Phe-Pro amide and pGlu-Glu-Pro amide increase plasma triiodothyronine levels in the mouse; the activity is sensitive to testosterone. Eur. J. Pharmacol. 358, 63–67. 10.1016/S0014-2999(98)00593-7 9809870

[B36] Cruz C. Charli J.-L. Vargas M. A. Joseph-Bravo P. (1991). Neuronal localization of pyroglutamate aminopeptidase II in primary cultures of fetal mouse brain. J. Neurochem. 56, 1594–1601. 10.1111/j.1471-4159.1991.tb02056.x 1672883

[B37] Cruz R. Vargas M. A. Uribe R. M. Pascual I. Lazcano I. Yiotakis A. (2008). Anterior pituitary pyroglutamyl peptidase II activity controls TRH-induced prolactin release. Peptides 29, 1953–1964. 10.1016/j.peptides.2008.07.011 18703099

[B38] Cucinotta D. Senin U. Girardello R. Crepaldi G. (1994). Posatirelin effect on patients with senile dementia of Alzheimer type (SDAT): a double-blind multicentre trial vs ascorbic acid and citicoline. J. Neurol. 241, S129.

[B39] Cushman D. W. Cheung H. S. Sabo E. F. Ondetti M. A. (1977). Design of potent competitive inhibitors of angiotensin-converting enzyme. Carboxyalkanoyl and mercaptoalkanoyl amino acids. Biochemistry 16, 5484–5491. 10.1021/bi00644a014 200262

[B40] Czekay G. Bauer K. (1993). Identification of the thyrotropin-releasing-hormone-degrading ectoenzyme as a metallopeptidase. Biochem. J. 290, 921–926. 10.1042/bj2900921 8096131 PMC1132368

[B41] Daimon C. Chirdon P. Maudsley S. Martin B. (2013). The role of Thyrotropin Releasing Hormone in aging and neurodegenerative diseases. AJAD 29–59. 10.7726/ajad.2013.1003 PMC3817016 24199031

[B42] de Gortari P. Fernández-Guardiola A. Martinez A. Cisneros M. Joseph-Bravo P. (1995). Changes in TRH and its degrading enzyme pyroglutamyl peptidase II, during the development of amygdaloid kindling. Brain Res. 679, 144–150. 10.1016/0006-8993(95)00237-K 7648256

[B43] de Gortari P. Romero F. Cisneros M. Joseph-bravo P. (2005). Acute administration of alcohol modulates pyroglutamyl amino peptidase II activity and mRNA levels in rat limbic regions. Neurochem. Int. 46, 347–356. 10.1016/j.neuint.2004.11.002 15707699

[B44] de Gortari P. Uribe R. M. García-Vázquez A. Aguilar-Valles A. Martínez A. Valdés A. (2006). Amygdala kindling differentially regulates the expression of the elements involved in TRH transmission. Neurochem. Int. 48, 31–42. 10.1016/j.neuint.2005.08.003 16213061

[B45] Deng P.-Y. Porter J. E. Shin H.-S. Lei S. (2006). Thyrotropin-releasing hormone increases GABA release in rat hippocampus: TRH modulation of hippocampal GABAergic transmission. J. Physiol. 577, 497–511. 10.1113/jphysiol.2006.118141 16990402 PMC1890442

[B46] Diano S. Naftolin F. Goglia F. Horvath T. L. (1998). Fasting-induced increase in type II iodothyronine deiodinase activity and messenger ribonucleic acid levels is not reversed by thyroxine in the rat hypothalamus. Endocrinology 139, 2879–2884. 10.1210/endo.139.6.6062 9607797

[B47] Drago F. Pulvirenti L. Spadaro F. Pennisi G. (1990). Effects of TRH and prolactin in the behavioral despair (swim) model of depression in rats. Psychoneuroendocrinology 15, 349–356. 10.1016/0306-4530(90)90060-M 2129310

[B48] Drago F. Coppi G. Antonuzzo P. A. Valerio C. Genazzani A. A. Grassi M. (1996). Effects of RGH 2202 on cognitive and motor behavior of the rat. Neurobiol. Aging 17, 67–71. 10.1016/0197-4580(95)02006-3 8786805

[B49] Elmore M. A. Griffiths E. C. O'Connor B. O'Cuinn G. (1990). Further characterization of the substrate specificity of a TRH hydrolysing pyroglutamate aminopeptidase from guinea-pig brain. Neuropeptides 15, 31–36. 10.1016/0143-4179(90)90157-T 1970134

[B50] Ensemble release 99 - January 2020. EMBL-EBI. http://Jan2020.archive.ensembl.org/Homo_sapiens/Gene/ExpressionAtlas?g=ENSG00000072657;r=12:72087266-72670758 [accessed march 21, 2020].

[B51] Faivre-Bauman A. Loudes C. Barret A. Tixier-Vidal A. Bauer K. (1986). Possible role of neuropeptide degrading enzymes on thyroliberin secretion in fetal hypothalamic cultures grown in serum free medium. Neuropeptides 7, 125–138. 10.1016/0143-4179(86)90088-0 2871517

[B52] Farre D. (2003). Identification of patterns in biological sequences at the ALGGEN server: PROMO and MALGEN. Nucleic Acids Res. 31, 3651–3653. 10.1093/nar/gkg605 12824386 PMC169011

[B53] Fehlings M. G. Baptiste D. C. (2005). Current status of clinical trials for acute spinal cord injury. Injury 36, S113–S122. 10.1016/j.injury.2005.06.022 15993112

[B54] Fekete C. Lechan R. M. (2014). Central regulation of hypothalamic-pituitary-thyroid axis under physiological and pathophysiological conditions. Endocrine Rev. 35, 159–194. 10.1210/er.2013-1087 24423980 PMC3963261

[B55] Ferguson J. F. Xue C. Hu Y. Li M. Reilly M. P. (2016). Adipose tissue RNASeq reveals novel gene–nutrient interactions following n-3 PUFA supplementation and evoked inflammation in humans. J. Nutr. Biochem. 30, 126–132. 10.1016/j.jnutbio.2015.12.010 27012629 PMC4808243

[B56] Finan B. Clemmensen C. Zhu Z. Stemmer K. Gauthier K. Müller L. (2016). Chemical hybridization of glucagon and thyroid hormone optimizes therapeutic impact for metabolic disease. Cell 167, 843–857.e14. 10.1016/j.cell.2016.09.014 27720451

[B57] Fröhlich E. Wahl R. (2019). The forgotten effects of thyrotropin-releasing hormone: Metabolic functions and medical applications. Front. Neuroendocrinol. 52, 29–43. 10.1016/j.yfrne.2018.06.006 29935915

[B58] Friedman T. C. Wilk S. (1985). The effect of inhibitors of prolyl endopeptidase and pyroglutamyl peptide hydrolase on TRH degradation in rat serum. Biochem. Biophys. Res. Commun. 132, 787–794. 10.1016/0006-291X(85)91201-X 2865955

[B59] Gallagher S. P. O'Connor B. (1998). A study of a highly specific pyroglutamyl aminopeptidase type-II from the membrane fraction of bovine brain. Int. J. Biochem. Cell Biol. 30, 115–133. 10.1016/S1357-2725(97)00074-5 9597758

[B60] Garat B. Miranda J. Charli J.-L. Ioseph-Bravo P. (1985). Presence of a membrane bound pyroglutamyl amino peptidase degrading thyrotropin releasing hormone in rat brain. Neuropeptides 6, 27–40. 10.1016/0143-4179(85)90128-3 2859545

[B61] Gary K. A. Sevarino K. A. Yarbrough G. G. Prange A. J. Winokur A. (2003). The Thyrotropin-Releasing Hormone (TRH) hypothesis of homeostatic regulation: implications for TRH-based therapeutics. J. Pharmacol. Exp. Ther. 305, 410–416. 10.1124/jpet.102.044040 12606661

[B62] Ghilchik M. W. Tobaruela M. del Rio-Garcia J. Smyth D. G. (2000). Characterization of neutral TRH-like peptides in mammary gland, mammary tumors and milk. Biochim. Biophys. Acta (BBA) Gen. Subj. 1475, 55–60. 10.1016/S0304-4165(00)00043-X 10806338

[B63] Gkonos P. J. Kwok C. K. Block N. L. Roos B. A. (1994). Identification of the human seminal TRH-like peptide pGlu-Phe-Pro-NH2 in normal human prostate. Peptides 15, 1281–1283. 10.1016/0196-9781(94)90154-6 7854981

[B64] González J. A. Horjales-Araujo E. Fugger L. Broberger C. Burdakov D. (2009). Stimulation of orexin/hypocretin neurones by thyrotropin-releasing hormone: TRH activates central orexin neurones. J. Physiol. 587, 1179–1186. 10.1113/jphysiol.2008.167940 19204048 PMC2674990

[B65] Gotoh K. Fukagawa K. Fukagawa T. Noguchi H. Kakuma T. Sakata T. (2007). Hypothalamic neuronal histamine mediates the thyrotropin-releasing hormone-induced suppression of food intake. J. Neurochem. 103, 1102–1110. 10.1111/j.1471-4159.2007.04802.x 17760865 PMC2156111

[B66] Grattan D. R. (2015). 60 YEARS OF NEUROENDOCRINOLOGY: The hypothalamo-prolactin axis. J. Endocrinol. 226, T101–T122. 10.1530/JOE-15-0213 26101377 PMC4515538

[B67] Griffiths E. C. McDermott J. R. Smith A. I. (1982). Mechanisms of brain inactivation of centrally-acting thyrotrophin-releasing hormone (TRH) analogues: a high-performance liquid chromatography study. Regul. Peptides 5, 1–11. 10.1016/0167-0115(82)90070-2 6820170

[B68] Gutiérrez-Mariscal M. de Gortari P. López-Rubalcava C. Martínez A. Joseph-Bravo P. (2008). Analysis of the anxiolytic-like effect of TRH and the response of amygdalar TRHergic neurons in anxiety. Psychoneuroendocrinology 33, 198–213. 10.1016/j.psyneuen.2007.11.002 18079066

[B69] Gutiérrez-Mariscal M. Sánchez E. Rebolledo-Solleiro D. García-Vázquez A. I. Cote-Vélez A. Acasuso-Rivero C. (2012). The acute response of the amygdalar TRH system to psychogenic stressors varies dependent on the paradigm and circadian condition. Brain Res. 1452, 73–84. 10.1016/j.brainres.2012.02.071 22464182

[B70] Hökfelt T. Tsuruo Y. Ulfhake B. Cullheim S. Arvidsson U. Foster G. A. (1989). SECTION II. SYNAPTIC ROLE OF TRH: Distribution of TRH-like immunoreactivity with special reference to coexistence with other neuroactive compounds. Ann. NY Acad. Sci. 553, 76–105. 10.1111/j.1749-6632.1989.tb46633.x 2497689

[B71] Hara J. Gerashchenko D. Wisor J. P. Sakurai T. Xie X. Kilduff T. S. (2009). Thyrotropin-Releasing Hormone increases behavioral arousal through modulation of hypocretin/orexin neurons. J. Neurosci. 29, 3705–3714. 10.1523/JNEUROSCI.0431-09.2009 19321767 PMC2712668

[B72] Heuer H. Ehrchen J. Bauer K. Schäfer M. K. H. (1998a). Region-specific expression of thyrotrophin-releasing hormone-degrading ectoenzyme in the rat central nervous system and pituitary gland: Distribution of the TRH-degrading ectoenzyme. Eur. J. Neurosci. 10, 1465–1478. 10.1046/j.1460-9568.1998.00158.x 9749801

[B73] Heuer H. Schäfer M. K.-H. Bauer K. (1998b). The Thyrotropin-Releasing Hormone-degrading ectoenzyme: the third element of the Thyrotropin-Releasing Hormone-signaling system. Thyroid 8, 915–920. 10.1089/thy.1998.8.915 9827659

[B74] Heuer H. Schäfer M. K. H. O'Donnell D. Walker P. Bauer K. (2000). Expression of thyrotropin-releasing hormone receptor 2 (TRH-R2) in the central nervous system of rats. J. Comp. Neurol. 428, 319–336. 10.1002/1096-9861(20001211)428:2<319::AID-CNE10>3.0.CO;2-9 11064370

[B75] Hicks S. C. Okrah K. Paulson J. N. Quackenbush J. Irizarry R. A. Bravo H. C. (2018). Smooth quantile normalization. Biostatistics 19, 185–198. 10.1093/biostatistics/kxx028 29036413 PMC5862355

[B76] Hinkle P. M. Pekary A. E. Senanayaki S. Sattin A. (2002). Role of TRH receptors as possible mediators of analeptic actions of TRH-like peptides. Brain Res. 935, 59–64. 10.1016/S0006-8993(02)02454-X 12062473

[B77] Hinkle P. M. Gehret A. U. Jones B. W. (2012). Desensitization, trafficking, and resensitization of the pituitary Thyrotropin-Releasing Hormone receptor. Front. Neurosci. 6, 180. 10.3389/fnins.2012.00180 23248581 PMC3521152

[B78] Hodges M. R. Richerson G. B. (2008). Contributions of 5-HT neurons to respiratory control: Neuromodulatory and trophic effects. Respiratory Physiol. Neurobiol. 164, 222–232. 10.1016/j.resp.2008.05.014 PMC2642893 18595785

[B79] Horita A. (1998). An update on the CNS actions of TRH and its analogs. Life Sci. 62, 1443–1448. 10.1016/S0024-3205(98)00087-3 9585116

[B80] Hou L. Zhou X. Chen Y. Qiu D. Zhu L. Wang J. (2012). Thyrotropin-releasing hormone causes a tonic excitatory postsynaptic current and inhibits the phasic inspiratory inhibitory inputs in inspiratory-inhibited airway vagal preganglionic neurons. Neuroscience 202, 184–191. 10.1016/j.neuroscience.2011.12.003 22198018

[B81] Ijiro T. Nakamura K. Ogata M. Inada H. Kiguchi S. Maruyama K. (2015). Effect of rovatirelin, a novel thyrotropin-releasing hormone analog, on the central noradrenergic system. Eur. J. Pharmacol. 761, 413–422. 10.1016/j.ejphar.2015.05.047 26142830

[B82] Itoh Y. Ogasawara T. Mushiroi T. Yamazaki A. Ukai Y. Kimura K. (1994). Effect of NS-3, a thyrotropin-releasing hormone analog, on in vivo acetylcholine release in rat brain: regional differences and its sites of action. J. Pharmacol. Exp. Ther. 271, 884–890. 7965809

[B83] Itoh Y. Sugimoto T. Ukai Y. Morino A. Kimura K. (1995). Permeability of NS-3, a Thyrotropin-releasing Hormone analogue, into the brain after its systemic administration in rats: a microdialysis study. J. Pharm. Pharmacol. 47, 833–836. 10.1111/j.2042-7158.1995.tb05750.x 8583352

[B84] Jaimes-Hoy L. Gutiérrez-Mariscal M. Vargas Y. Pérez-Maldonado A. Romero F. Sánchez-Jaramillo E. (2016). Neonatal maternal separation alters, in a sex-specific manner, the expression of TRH, of TRH-degrading ectoenzyme in the rat hypothalamus, and the response of the thyroid axis to starvation. Endocrinology 157, 3253–3265. 10.1210/en.2016-1239 27323240

[B85] Jaimes-Hoy L. Romero F. Charli J.-L. Joseph-Bravo P. (2019). Sex dimorphic responses of the hypothalamus–pituitary–thyroid axis to maternal separation and palatable diet. Front. Endocrinol. 10, 445. 10.3389/fendo.2019.00445 PMC6637657 31354623

[B86] Jeong J. K. Szabo G. Kelly K. Diano S. (2012). Prolyl carboxypeptidase regulates energy expenditure and the thyroid axis. Endocrinology 153, 683–689. 10.1210/en.2011-1399 22202165 PMC3275392

[B87] Jin H. Fedorowicz G. Yang R. Ogasawara A. Peale F. Pham T. (2004). Thyrotropin-Releasing Hormone is induced in the left ventricle of rats with heart failure and can provide inotropic support to the failing heart. Circulation 109, 2240–2245. 10.1161/01.CIR.0000127951.13380.B4 15096458

[B88] Joseph-Bravo P. Fresán M. E. Cisneros M. Vargas M. A. Charli J.-L. (1994). Pyroglutamyl peptidase II activity is not in the processes of bulbospinal TRHergic neurons. Neurosci. Lett. 178, 243–246. 10.1016/0304-3940(94)90769-2 7824205

[B89] Joseph-Bravo P. Jaimes-Hoy L. Charli J.-L. (2015a). Regulation of TRH neurons and energy homeostasis-related signals under stress. J. Endocrinol. 224, R139–R159. 10.1530/JOE-14-0593 25563352

[B90] Joseph-Bravo P. Jaimes-Hoy L. Uribe R.-M. Charli J.-L. (2015b). 60 YEARS OF NEUROENDOCRINOLOGY: TRH, the first hypophysiotropic releasing hormone isolated: control of the pituitary–thyroid axis. J. Endocrinol. 226, T85–T100. 10.1530/JOE-15-0124 26101376

[B91] Kalivas P. W. Stanley D. Prange A. J. (1987). Interaction between thyrotropinreleasing hormone and the mesolimbic dopamine system. Neuropharmacology 26, 33–38. 10.1016/0028-3908(87)90041-4 3104814

[B92] Kamath J. Yarbrough G. G. Prange A. J. Winokur A. (2009). The thyrotropin-releasing hormone (TRH)–immune system homeostatic hypothesis. Pharmacol. Ther. 121, 20–28. 10.1016/j.pharmthera.2008.09.004 19000920

[B93] Kanasaki H. Oride A. Mijiddorj T. Purwana I. Miyazaki K. (2011). Secondary amenorrhea in a woman with spinocerebellar degeneration treated with thyrotropin-releasing hormone: a case report and in vitro analysis. J. Med. Case Rep. 5, 567. 10.1186/1752-1947-5-567 22152284 PMC3261233

[B94] Kaysser L. (2019). Built to bind: biosynthetic strategies for the formation of small-molecule protease inhibitors. Nat. Prod. Rep. 36, 1654–1686. 10.1039/C8NP00095F 30976762

[B95] Kelly J. A. Loscher C. E. Gallagher S. O'Connor B. (1997). Degradation of pyroglutamyl-phenylalanyl-proline amide by a pyroglutamyl aminopeptidase purified from membrane fractions of bovine brain. Biochem. Soc. Trans. 25, 114S–114S. 10.1042/bst025114s 9057012

[B96] Kelly J. A. Slator G. R. Tipton K. F. Williams C. H. Bauer K. (2000). Kinetic investigation of the specificity of porcine brain Thyrotropin-releasing Hormone-degrading ectoenzyme for Thyrotropin-releasing Hormone-like peptides. J. Biol. Chem. 275, 16746–16751. 10.1074/jbc.M910386199 10748219

[B97] Kelly J. A. Scalabrino G. A. Slator G. R. Cullen A. A. Gilmer J. F. Lloyd D. G. (2005). Structure–activity studies with high-affinity inhibitors of pyroglutamyl-peptidase II. Biochem. J. 389, 569–576. 10.1042/BJ20041722 15799721 PMC1175135

[B98] Kelly J. A. Boyle N. T. Cole N. Slator G. R. Colivicchi M. A. Stefanini C. (2015). First-in-class thyrotropin-releasing hormone (TRH)-based compound binds to a pharmacologically distinct TRH receptor subtype in human brain and is effective in neurodegenerative models. Neuropharmacology 89, 193–203. 10.1016/j.neuropharm.2014.09.024 25281210

[B99] Kelly J. A. (1995). Thyrotropin-releasing hormone: basis and potential for its therapeutic use. Essays Biochem. 30, 133–149. 8822153

[B100] Khan Z. Aitken A. Garcia J. R. Smyth D. G. (1992). Isolation and identification of two neutral thyrotropin releasing hormone-like peptides, pyroglutamylphenylalanineproline amide and pyroglutamylglutamineproline amide, from human seminal fluid. J. Biol. Chem. 267, 7464–7469. 1559984

[B101] Khomane K. S. Meena C. L. Jain R. Bansal A. K. (2011). Novel thyrotropin-releasing hormone analogs: a patent review. Expert Opin. Ther. Patents 21, 1673–1691. 10.1517/13543776.2011.623127 22017410

[B102] Kinoshita K. Nagao T. Ono H. (1994). Effects of TA-0910, an orally active TRH analog, on the spinal reflex in spinal rats. Neuropharmacology 33, 1183–1188. 10.1016/S0028-3908(05)80008-5 7862253

[B103] Kinoshita K. Yamamura M. Sugihara J. Suzuki M. Matsuoka Y. (1998). Taltirelin Hydrate (TA-0910): an orally active Thyrotropin-Releasing Hormone mimetic agent with multiple actions. CNS Drug Rev. 4, 25–41. 10.1111/j.1527-3458.1998.tb00039.x

[B104] Kobayashi N. Sato N. Fujimura Y. Kihara T. Sugita K. Takahashi K. (2018). Discovery of the orally effective Thyrotropin-Releasing Hormone mimetic: 1-{*N* -[(4 *S* ,5 *S* )-(5-Methyl-2-oxooxazolidine-4-yl)carbonyl]-3-(thiazol-4-yl)- l -alanyl}-(2 *R* )-2-methylpyrrolidine Trihydrate (Rovatirelin Hydrate). ACS Omega 3, 13647–13666. 10.1021/acsomega.8b01481 30411045 PMC6217654

[B105] Kobayashi K. Abe Y. Harada H. Oota E. Endo T. Takeda H. (2019a). Non-clinical pharmacokinetic profiles of rovatirelin, an orally available thyrotropin-releasing hormone analogue. Xenobiotica 49, 106–119. 10.1080/00498254.2017.1423130 29300135

[B106] Kobayshi K. Abe Y. Kawai A. Furihata T. Harada H. Endo T. (2019b). Human mass balance, pharmacokinetics and metabolism of rovatirelin and identification of its metabolic enzymes *in vitro*. Xenobiotica 49, 1434–1446. 10.1080/00498254.2019.1580796 30747023

[B107] Kodama H. Furuuchi S. Takahashi M. Sugihara J. Yoshikawa M. (1997). Disposition of Taltirelin. (1): absorption, distribution, metabolism and excretion in rats and dogs. Drug Metab. Pharmacokinet. 12, 460–474. 10.2133/dmpk.12.460

[B108] Kubek M. J. Low W. C. Sattin A. Morzorati S. L. Meyerhoff J. L. Larsen S. H. (1989). Role of TRH in seizure modulation. Ann. N. Y. Acad. Sci. 553, 286–303. 10.1111/j.1749-6632.1989.tb46650.x 2470309

[B109] Kubek M. J. Knoblach S. M. Sharif N. A. Burt D. R. Buterbaugh G. G. Fuson K. S. (1993). Thyrotropin-Releasing hormone gene expression and receptors are differentially modified in limbic foci by seizures. Ann. Neurol. 33, 70–76. 10.1002/ana.410330112 8388190

[B110] Kulkarni R. N. Wang Z. L. Akinsanya K. O. Bennet W. M. Wang R. M. Smith D. M. (1995). Pyroglutamyl-phenylalanyl-proline amide attenuates thyrotropin-releasing hormone-stimulated insulin secretion in perifused rat islets and insulin-secreting clonal beta-cell lines. Endocrinology 136, 5155–5164. 10.1210/endo.136.11.7588254 7588254

[B111] Lai C.-T. Li H.-J. Yu W. Shah S. Bommineni G. R. Perrone V. (2015). Rational modulation of the induced-fit conformational change for slow-onset inhibition in *Mycobacterium tuberculosis* InhA. Biochemistry 54, 4683–4691. 10.1021/acs.biochem.5b00284 26147157 PMC4805124

[B112] Lanzara R. Liebman M. Wilk S. (1989). The use of analogues of TRH to probe the specificity of pyroglutamyl peptidase II. Ann. NY Acad. Sci. 553, 559–562. 10.1111/j.1749-6632.1989.tb46696.x

[B113] Lazcano I. Uribe R. M. Martínez-Chávez E. Vargas M. A. Matziari M. Joseph-Bravo P. (2012). Pyroglutamyl peptidase II inhibition enhances the analeptic effect of Thyrotropin-Releasing Hormone in the rat medial septum. J. Pharmacol. Exp. Ther. 342, 222–231. 10.1124/jpet.112.192278 22532627

[B114] Lazcano I. Cabral A. Uribe R. M. Jaimes-Hoy L. Perello M. Joseph-Bravo P. (2015). Fasting enhances pyroglutamyl peptidase II activity in tanycytes of the mediobasal hypothalamus of male adult rats. Endocrinology 156, 2713–2723. 10.1210/en.2014-1885 25942072

[B115] Lechan R. M. Wu P. Jackson I. M. D. (1986). Immunolocalization of the Thyrotropin-Releasing Hormone prohormone in the rat central nervous system. Endocrinology 119, 1210–1216. 10.1210/endo-119-3-1210 3089766

[B116] Leppäluoto J. Koivusalo F. Kraama R. (1978). Thyrotropin-releasing factor: Distribution in neural and gastrointestinal tissues. Acta Physiol. Scandinavica 104, 175–179. 10.1111/j.1748-1716.1978.tb06264.x 102111

[B117] Lesnikov V. A. Korneva E. A. Dall'ara A. Pierpaoli W. (1992). The involvement of pineal gland and melatonin in immunity and aging: II. Thyrotropin-Releasing Hormone and melatonin forestall involution and promote reconstitution of the thymus in anterior hypothalamic area (Aha)-lesioned mice. Int. J. Neurosci. 62, 141–153. 10.3109/00207459108999767 1342010

[B118] Lin J. Wilk S. (1998). Quantitation and regulation of pyroglutamyl peptidase II messenger RNA levels in rat tissues and GH3 cells. Neuroendocrinology 67, 197–208. 10.1159/000054315 9630437

[B119] Linden H. del Rio Garcia J. Huber A. Kreil G. Smyth D. (1996). The TRH-like peptides in rabbit testis are different from the TRH-like peptide in the prostate. FEBS Lett. 379, 11–14. 10.1016/0014-5793(95)01468-3 8566220

[B120] Luo L. Yano N. (2004). Expression of thyrotropin-releasing hormone receptor in immortalized beta-cell lines and rat pancreas. J. Endocrinol. 181, 401–412. 10.1677/joe.0.1810401 15171688

[B121] Méndez M. Cisneros M. Baez A. Joseph-Bravo P. Charli J. L. (1999). Three TRH-like molecules are released from rat hypothalamus in vitro. Neurochem. Res. 24, 815–823. 10.1023/a:1020993527602 10403620

[B122] Müller-Fielitz H. Stahr M. Bernau M. Richter M. Abele S. Krajka V. (2017). Tanycytes control the hormonal output of the hypothalamic-pituitary-thyroid axis. Nat. Commun. 8, 484. 10.1038/s41467-017-00604-6 28883467 PMC5589884

[B123] Marsili A. Sanchez E. Singru P. Harney J. W. Zavacki A. M. Lechan R. M. (2011). Thyroxine-induced expression of pyroglutamyl peptidase II and inhibition of TSH release precedes suppression of TRH mRNA and requires type 2 deiodinase. J. Endocrinol. 211, 73–78. 10.1530/JOE-11-0248 21788297 PMC3558748

[B124] Martinez de la Escalera G. Weiner R. I. (1992). Dissociation of dopamine from its receptor as a signal in the pleiotropic hypothalamic regulation of prolactin secretion. Endocr. Rev. 13, 241–255. 10.1210/edrv-13-2-241 1618163

[B125] Martino E. Lernmark A. Seo H. Steiner D. F. Refetoff S. (1978). High concentration of thyrotropin-releasing hormone in pancreatic islets. Proc. Natl. Acad. Sci. 75, 4265–4267. 10.1073/pnas.75.9.4265 100783 PMC336093

[B126] Matre V. Høvring P. I. Fjeldheim Å.-K. Helgeland L. Orvain C. Andersson K. B. (2003). The human neuroendocrine thyrotropin-releasing hormone receptor promoter is activated by the haematopoietic transcription factor c-Myb. Biochem. J. 372, 851–859. 10.1042/bj20030057 12628004 PMC1223435

[B127] Matziari M. Bauer K. Dive V. Yiotakis A. (2008). Synthesis of the phosphinic analogue of Thyrotropin Releasing Hormone. J. Org. Chem. 73, 8591–8593. 10.1021/jo8014215 18826326

[B128] Meena C. L. Ingole S. Rajpoot S. Thakur A. Nandekar P. P. Sangamwar A. T. (2015). Discovery of a low affinity thyrotropin-releasing hormone (TRH)-like peptide that exhibits potent inhibition of scopolamine-induced memory impairment in mice. RSC Adv. 5, 56872–56884. 10.1039/C5RA06935A 26191403 PMC4501038

[B129] Mentlein R. (1999). Dipeptidyl-peptidase IV (CD26)-role in the inactivation of regulatory peptides. Regul. Peptides 85, 9–24. 10.1016/S0167-0115(99)00089-0 10588446

[B130] Messeguer X. Escudero R. Farre D. Nunez O. Martinez J. Alba M. M. (2002). PROMO: detection of known transcription regulatory elements using species-tailored searches. Bioinformatics 18, 333–334. 10.1093/bioinformatics/18.2.333 11847087

[B131] Minelli A. Bellezza I. Grottelli S. Galli F. (2008). Focus on cyclo(His-Pro): history and perspectives as antioxidant peptide. Amino Acids 35, 283–289. 10.1007/s00726-007-0629-6 18163175

[B132] Mitsuma T. Rhue N. Sobue G. Hirooka Y. Kayama M. Yokoi Y. (1995). Distribution of thyrotropin releasing hormone receptor in rats: an immunohistochemical study. Endocr. Regul. 29, 129–134. 10993974

[B133] Monga V. Meena C. Kaur N. Jain R. (2008). Chemistry and biology of Thyrotropin-Releasing Hormone (TRH) and its analogs. CMC 15, 2718–2733. 10.2174/092986708786242912 18991632

[B134] Montagne J.-J. Ladram A. Nicolas P. Bulant M. (1999). Cloning of Thyrotropin-Releasing Hormone precursor and receptor in rat thymus, adrenal gland, and testis. Endocrinology 140, 1054–1059. 10.1210/endo.140.3.6558 10067825

[B135] Morier E. Moreau O. Masson M. A. Han K.-K. Rips R. (1979). Evidence for the enzymic degradation of thyrotropin-releasing-hormone (trh) and pseudo-hormone (pyroglutamyl-histidyl-amphetamine) by calf liver pyroglutamine-amino-peptidase. Int. J. Biochem. 10, 769–783. 10.1016/0020-711X(79)90155-1 39805

[B136] Morley J. E. (1979). Extrahypothalamic Thyrotropin Releasing Hormone (TRH) — Its distribution and its functions. Life Sci. 25, 1539–1550. 10.1016/0024-3205(79)90435-1 118318

[B137] Mulla C. M. Geras-Raaka E. Raaka B. M. Gershengorn M. C. (2009). High levels of Thyrotropin-Releasing Hormone receptors activate programmed cell death in human pancreatic precursors. Pancreas 38, 197–202. 10.1097/MPA.0b013e31818d14a8 18948837 PMC2647589

[B138] Nakamura T. Honda M. Kimura S. Tanabe M. Oda S. Ono H. (2005). Taltirelin improves motor ataxia independently of monoamine levels in rolling mouse Nagoya, a model of spinocerebellar atrophy. Biol. Pharm. Bull. 28, 2244–2247. 10.1248/bpb.28.2244 16327158

[B139] Nishio Y. Kusugami K. Kaneko H. Yamamoto H. Konagaya T. Nagai H. (1999). Intraluminal thyrotropin-releasing hormone affects gastric somatostatin and acid secretion through its specific receptor in rats. Scand. J. Gastroenterol. 34, 270–275. 10.1080/00365529950173672 10232871

[B140] O'Connor B. O'Cuinn G. (1985). Purification of and kinetic studies on a narrow specificity synaptosomal membrane pyroglutamate aminopeptidase from guinea-pig brain. Eur. J. Biochem. 150, 47–52. 10.1111/j.1432-1033.1985.tb08986.x 2862039

[B141] O'Leary R. O'Connor B. (1995). Thyrotropin-releasing hormone. J. Neurochem. 65, 953–963. 10.1046/j.1471-4159.1995.65030953.x 7643125

[B142] Ogawa N. Mizuno S. Nukina I. Tsukamoto S. Mori A. (1983). Chronic thyrotropin releasing hormone (TRH) administration on TRH receptors and muscarinic cholinergic receptors in CNS. Brain Res. 263, 348–350. 10.1016/0006-8993(83)90328-1 6301651

[B143] Oka M. Ochi Y. Furukawa K. Ito T. Miura Y. Karasawa T. (1989). L-6-ketopiperidine-2-carbonyl-L-leucyl-L-proline amide as a novel thyrotropin releasing hormone analogue with improving effects on impaired central nervous systems functions. Arzneimittelforschung 39, 297–303. 2547386

[B144] Péterfi Z. Farkas E. Nagyunyomi-Sényi K. Kádár A. Ottó S. Horváth A. (2018). Role of TRH/UCN3 neurons of the perifornical area/bed nucleus of stria terminalis region in the regulation of the anorexigenic POMC neurons of the arcuate nucleus in male mice and rats. Brain Struct. Funct. 223, 1329–1341. 10.1007/s00429-017-1553-5 29124350

[B145] Parnetti L. Ambrosoli L. Abate G. Azzini C. Balestreri R. Bartorelli L. (1995). Posatirelin for the treatment of late-onset Alzheimer's disease: a double-blind multicentre study vs citicoline and ascorbic acid. Acta Neurol. Scand. 92, 135–140. 10.1111/j.1600-0404.1995.tb01027.x 7484061

[B146] Pascual I. Gil-Parrado S. Cisneros M. Joseph-Bravo P. Díaz J. Possani L. D. (2004). Purification of a specific inhibitor of pyroglutamyl aminopeptidase II from the marine annelide Hermodice carunculata. Int. J. Biochem. Cell Biol. 36, 138–152. 10.1016/S1357-2725(03)00175-4 14592539

[B147] Patchett A. A. Cordes E. H. (1985). “The design and properties of N-Carboxyalkyldipeptide inhibitors of angiotensin-converting Enzyme,” in Advances in Enzymology - and Related Areas of Molecular Biology. Ed. Meister A. (Hoboken, NJ, USA: John Wiley & Sons, Inc.), 1–84. 10.1002/9780470123034.ch1 2994404

[B148] Pawlikowski M. Żerek-Mełeń G. Winczyk K. (1992). Thyroliberin (TRH) increases thymus cell proliferation in rats. Neuropeptides 23, 199–202. 10.1016/0143-4179(92)90123-E 1470310

[B149] Pekary A. E. Sattin A. (2012). Rapid modulation of TRH and TRH-like peptide release in rat brain and peripheral tissues by ghrelin and 3-TRP-ghrelin. Peptides 36, 157–167. 10.1016/j.peptides.2012.04.021 22634385

[B150] Pekary A. E. Sattin A. (2017). TRH and TRH-Like peptide levels co-vary with reproductive and metabolic rhythms. Horm. Metab. Res. 49, 86–94. 10.1055/s-0042-111012 27434852

[B151] Pekary A. E. Sattin A. Meyerhoff J. L. Chilingar M. (2004). Valproate modulates TRH receptor, TRH and TRH-like peptide levels in rat brain. Peptides 25, 647–658. 10.1016/j.peptides.2004.01.016 15165721

[B152] Pekary A. E. Faull K. F. Paulson M. Lloyd R. L. Sattin A. (2005). TRH-like antidepressant peptide, pyroglutamyltyroslyprolineamide, occurs in rat brain. J. Mass Spectrom 40, 1232–1236. 10.1002/jms.904 16124040

[B153] Pekary A. E. Sattin A. Lloyd R. L. (2015). Ketamine modulates TRH and TRH-like peptide turnover in brain and peripheral tissues of male rats. Peptides 69, 66–76. 10.1016/j.peptides.2015.04.003 25882008

[B154] Peres Diaz L. S. Schuman M. L. Aisicovich M. Toblli J. E. Pirola C. J. Landa M. S. (2018). Angiotensin II requires an intact cardiac thyrotropin-releasing hormone (TRH) system to induce cardiac hypertrophy in mouse. J. Mol. Cell. Cardiol. 124, 1–11. 10.1016/j.yjmcc.2018.09.009 30267750

[B155] Perez Castro C. Peñalva R. Páez Pereda M. Renner U. Reul J. M. Stalla G. K. (1999). Early activation of thyrotropin-releasing-hormone and prolactin plays a critical role during a T cell-dependent immune response. Endocrinology 140, 690–697. 10.1210/endo.140.2.6482 9927295

[B156] Pierpaoli W. Yi C. (1990). The involvement of pineal gland and melatonin in immunity and aging I. Thymus-mediated, immunoreconstituting and antiviral activity of thyrotropin-releasing hormone. J. Neuroimmunol. 27, 99–109. 10.1016/0165-5728(90)90059-V 2159021

[B157] Pierpaoli W. (2013). Aging-reversing properties of Thyrotropin-Releasing Hormone. CAS 6, 92–98. 10.2174/1874609811306010012 23895526

[B158] Pilowsky P. M. (2014). ““Peptides, serotonin, and breathing,”,” in Progress in Brain Research (Oxford, UK:Elsevier), 169–189. 10.1016/B978-0-444-63274-6.00009-6 24746048

[B159] Prasad C. Jayaraman A. (1986). Metabolism of thyrotropin-releasing hormone in human cerebrospinal fluid. Isolation and characterization of pyroglutamate aminopeptidase activity. Brain Res. 364, 331–337. 10.1016/0006-8993(86)90845-0 2868782

[B160] Prasad C. Peterkofsky A. (1976). Demonstration of pyroglutamylpeptidase and amidase activities toward thyrotropin-releasing hormone in hamster hypothalamus extracts. J. Biol. Chem. 251, 3229–3234. 819429

[B161] Prasad C. (1989). Neurobiology of Cyclo(His-Pro). Ann. NY Acad. Sci. 553, 232–251. 10.1111/j.1749-6632.1989.tb46646.x 2541650

[B162] Prasad C. (1995). Bioactive cyclic dipeptides. Peptides 16, 151–164. 10.1016/0196-9781(94)00017-Z 7716068

[B163] Prokai-Tatrai K. Prokai L. (2009). Prodrugs of Thyrotropin-Releasing Hormone and related peptides as central nervous system agents. Molecules 14, 633–654. 10.3390/molecules14020633 19214153 PMC6253886

[B164] Puga L. Alcántara-Alonso V. Coffeen U. Jaimes O. de Gortari P. (2016). TRH injected into the nucleus accumbens shell releases dopamine and reduces feeding motivation in rats. Behav. Brain Res. 306, 128–136. 10.1016/j.bbr.2016.03.031 27006143

[B165] Rabeler R. Mittag J. Geffers L. Rüther U. Leitges M. Parlow A. F. (2004). Generation of Thyrotropin-Releasing Hormone receptor 1-deficient mice as an animal model of central hypothyroidism. Mol. Endocrinol. 18, 1450–1460. 10.1210/me.2004-0017 14988432

[B166] Rausell V. Fraser H. M. Tobaruela M. del Rio-Garcia J. Smyth D. G. (1999). Identification of the TRH-like peptides pGlu–Glu–Pro amide and pGlu–Phe–Pro amide in rat thyroid: regulation by thyroid status. Regul. Peptides 81, 55–60. 10.1016/S0167-0115(99)00017-8 10395408

[B167] Redding T. W. Schally A. V. (1969). Studies on the Thyrotropin-Releasing Hormone (TRH) activity in peripheral blood. Exp. Biol. Med. 131, 420–425. 10.3181/00379727-131-33892 4977749

[B168] Rodríguez-Molina V. Vargas M.Á. Joseph-Bravo P. Charli J.-L. (2009). NMDA receptor up-regulates pyroglutamyl peptidase II activity in the rat hippocampus. Neurosci. Lett. 449, 211–214. 10.1016/j.neulet.2008.11.005 19013213

[B169] Rodríguez-Molina V. Patiño J. Vargas Y. Sánchez-Jaramillo E. Joseph-Bravo P. Charli J.-L. (2014). TRH regulates action potential shape in cerebral cortex pyramidal neurons. Brain Res. 1571, 1–11. 10.1016/j.brainres.2014.05.015 24842001

[B170] Rodríguez-Rodríguez A. Lazcano I. Sánchez-Jaramillo E. Uribe R. M. Jaimes-Hoy L. Joseph-Bravo P. (2019). Tanycytes and the control of Thyrotropin-Releasing Hormone flux into portal capillaries. Front. Endocrinol. 10, 401. 10.3389/fendo.2019.00401 PMC6603095 31293518

[B171] Rondeel J. M. M. Klootwijk W. Linkels E. Jeucken W. H. M P. Hofland L. J. (1995a). Further studies on the regulation, localization and function of the TRH-like peptide pyroglutamyl-glutamyl-prolineamide in the rat anterior pituitary gland. J. Endocrinol. 146, 293–300. 10.1677/joe.0.1460293 7561642

[B172] Rondeel J. M. M. Klootwijk W. Linkels E. van Haasteren G. A. C. de Greef W. J. Visser T. J. (1995b). Regulation of the TRH-like peptide pyroglutamyl-glutamyl-prolineamide in the rat anterior pituitary gland. J. Endocrinol. 145, 43–49. 10.1677/joe.0.1450043 7798029

[B173] Rosen J. B. Cain C. J. Weiss S. R. B. Post R. M. (1992). Alterations in mRNA of enkephalin, dynorphin and thyrotropin releasing hormone during amygdala kindling: an in situ hybridization study. Mol. Brain Res. 15, 247–255. 10.1016/0169-328X(92)90115-R 1359374

[B174] Runfola M. Sestito S. Bellusci L. La Pietra V. D'Amore V. M. Kowalik M. A. (2020). Design, synthesis and biological evaluation of novel TRβ selective agonists sustained by ADME-toxicity analysis. Eur. J. Med. Chem. 188, 112006. 10.1016/j.ejmech.2019.112006 31931337

[B175] Sánchez E. Uribe R. M. Corkidi G. Zoeller R. T. Cisneros M. Zacarias M. (2001). Differential responses of Thyrotropin-Releasing Hormone (TRH) neurons to cold exposure or suckling indicate functional heterogeneity of the TRH system in the paraventricular nucleus of the rat hypothalamus. Neuroendocrinology 74, 407–422. 10.1159/000054707 11752897

[B176] Sánchez E. Vargas M. A. Singru P. S. Pascual I. Romero F. Fekete C. (2009). Tanycyte Pyroglutamyl Peptidase II contributes to regulation of the hypothalamic-pituitary-thyroid axis through glial-axonal associations in the median eminence. Endocrinology 150, 2283–2291. 10.1210/en.2008-1643 19179432 PMC2671897

[B177] Sánchez E. Charli J.-L. Lechan R. M. (2013). ““Pyroglutamyl-Peptidase II,”,” in Handbook of Proteolytic Enzymes (Oxford, UK:Elsevier), 414–419. 10.1016/B978-0-12-382219-2.00083-1

[B178] Sárvári A. Farkas E. Kádár A. Zséli G. Füzesi T. Lechan R. M. (2012). Thyrotropin-releasing hormone-containing axons innervate histaminergic neurons in the tuberomammillary nucleus. Brain Res. 1488, 72–80. 10.1016/j.brainres.2012.10.010 23063458 PMC3501586

[B179] Sah N. Rajput S. K. Singh J. N. Meena C. L. Jain R. Sikdar S. K. (2011). l-pGlu-(2-propyl)-l-His-l-ProNH2 attenuates 4-aminopyridine-induced epileptiform activity and sodium current: a possible action of new thyrotropin-releasing hormone analog for its anticonvulsant potential. Neuroscience 199, 74–85. 10.1016/j.neuroscience.2011.10.008 22037285

[B180] Saito Y. Mekuchi M. Kobayashi N. Kimura M. Aoki Y. Masuda T. (2011). Molecular cloning, molecular evolution and gene expression of cDNAs encoding thyrotropin-releasing hormone receptor subtypes in a teleost, the sockeye salmon (Oncorhynchus nerka). Gen. Comp. Endocrinol. 174, 80–88. 10.1016/j.ygcen.2011.07.011 21827760

[B181] Sasahara H. Otaka M. Itoh S. Iwabuchi A. Odashima M. Wada I. (1998). Effect of preinduction of heat-shock proteins on acetic acid-induced small intestinal lesions in rats. Dig. Dis. Sci. 43, 2117–2130. 10.1023/a:1018827802462 9753281

[B182] Sasaki I. Fujita T. Murakami M. Yamamoto A. Nakamura E. Imasaki H. (1994). Intestinal absorption of azetirelin, a new Thyrotropin-Releasing Hormone (TRH) analogue. I. Possible factors for the low oral bioavailability in rats. Biol. Pharm. Bull. 17, 1256–1261. 10.1248/bpb.17.1256 7841949

[B183] Sasaki I. Tozaki H. Matsumoto K. Ito Y. Fujita T. Murakami M. (1999). Development of an oral formulation of azetirelin, a new thyrotropin-releasing hormone (TRH) analogue, using n-lauryl-beta-D-maltopyranoside as an absorption enhancer. Biol. Pharm. Bull. 22, 611–615. 10.1248/bpb.22.611 10408236

[B184] Sattin A. (1999). The role of TRH and related peptides in the mechanism of action of ECT. J. ECT 15, 76–92. 10.1097/00124509-199903000-00007 10189620

[B185] Scalabrino G. A. Hogan N. O'Boyle K. M. Slator G. R. Gregg D. J. Fitchett C. M. (2007). Discovery of a dual action first-in-class peptide that mimics and enhances CNS-mediated actions of thyrotropin-releasing hormone. Neuropharmacology 52, 1472–1481. 10.1016/j.neuropharm.2007.02.003 17418282

[B186] Schauder B. Schomburg L. Kohrle J. Bauer K. (1994). Cloning of a cDNA encoding an ectoenzyme that degrades thyrotropin-releasing hormone. Proc. Natl. Acad. Sci. 91, 9534–9538. 10.1073/pnas.91.20.9534 7937801 PMC44847

[B187] Schmitmeier S. Thole H. Bader A. Bauer K. (2002). Purification and characterization of the thyrotropin-releasing hormone (TRH)-degrading serum enzyme and its identification as a product of liver origin. Eur. J. Biochem. 269, 1278–1286. 10.1046/j.1432-1033.2002.02768.x 11856362

[B188] Schomburg L. Turwitt S. Prescher G. Lohmann D. Horsthemke B. Bauer K. (1999). Human TRH-degrading ectoenzyme cDNA cloning, functional expression, genomic structure and chromosomal assignment. Eur. J. Biochem. 265, 415–422. 10.1046/j.1432-1327.1999.00753.x 10491199

[B189] Schuman M. L. Landa M. S. Toblli J. E. Peres Diaz L. S. Alvarez A. L. Finkielman S. (2011). Cardiac Thyrotropin-Releasing Hormone mediates left ventricular hypertrophy in spontaneously hypertensive rats. Hypertension 57, 103–109. 10.1161/HYPERTENSIONAHA.110.161265 21135357

[B190] Schuman M. L. Peres Diaz L. S. Landa M. S. Toblli J. E. Cao G. Alvarez A. L. (2014). Thyrotropin-releasing hormone overexpression induces structural changes of the left ventricle in the normal rat heart. Am. J. Physiol. Heart Circ. Physiol. 307, H1667–H1674. 10.1152/ajpheart.00494.2014 25281568

[B191] Segerson T. P. Hoefler H. Childers H. Wolfe H. J. Wu P. Jackson I. M. D. (1987). Localization of Thyrotropin-Releasing Hormone prohormone messenger ribonucleic acid in rat brain by in situ hybridization. Endocrinology 121, 98–107. 10.1210/endo-121-1-98 3109882

[B192] Sharif N. A. Towle A. C. Burt D. R. Mueller R. A. Breese G. R. (1989). Contransmitters: differential effects of serotonin (5-HT)-depleting drugs on levels of 5-HT and TRH and their receptors in rat brain and spinal cord. Brain Res. 480, 365–371. 10.1016/0006-8993(89)90209-6 2540880

[B193] Shi Z.-X. Xu W. Mergner W. J. Li Q.-L. Cole K. H. Wilber J. F. (1996). Localization of Thyrotropin-Releasing Hormone mRNA expression in the rat heart by in situ hybridization histochemistry. Pathobiology 64, 314–319. 10.1159/000164066 9159025

[B194] Shishido Y. Furushiro M. Tanabe S. Shibata S. Hashimoto S. Yokokura T. (1999). Effects of prolyl endopeptidase inhibitors and neuropeptides on delayed neuronal death in rats. Eur. J. Pharmacol. 372, 135–142. 10.1016/S0014-2999(99)00185-5 10395093

[B195] Simasko S. M. Horita A. (1985). Treatment of rats with the TRH analog MK-771. Neuropharmacology 24, 157–165. 10.1016/0028-3908(85)90175-3 2986033

[B196] Siviter R. J. Cockle S. M. (1995). Peptides related to thyrotrophin-releasing hormone are degraded in seminal plasma by an enzyme similar to prolyl endopeptidase. J. Endocrinol. 144, 61–66. 10.1677/joe.0.1440061 7891026

[B197] Sjöström H. Norén O. Olsen J. (2002). ““Structure and function of aminopeptidase N,”,” in Cellular Peptidases in Immune Functions and Diseases 2. Eds. Langner J. Ansorge S. ((Boston, MA: Springer US)), 25–34. 10.1007/0-306-46826-3_2

[B198] Smyth D. G. del Rio-Garcia J. Wallnöfer H. Gogl H. Simma W. Huber A. (1999). Protirelin (thyrotropin-releasing hormone) in thyroid gland: possible involvement in regulation of thyroid status. Zhongguo Yao Li Xue Bao 20, 289–291. 10452110

[B199] Štrbák V. (2018). Pancreatic Thyrotropin Releasing Hormone and Mechanism of Insulin Secretion. Cell Physiol. Biochem. 50, 378–384. 10.1159/000494013 30286449

[B200] Sugimoto T. Hayashi T. Okita A. Morino A. (1996). Pharmacokinetics of the new thyrotropin releasing hormone analogue montirelin hydrate. 1st communication: plasma concentrations, metabolism and excretion after a single intravenous administration to rats, dogs and monkeys. Arzneimittelforschung 46, 106–113. 8720298

[B201] Sun Y. Lu X. Gershengorn M. (2003). Thyrotropin-releasing hormone receptors – similarities and differences. J. Mol. Endocrinol. 30, 87–97. 10.1677/jme.0.0300087 12683933

[B202] Sun Y. Zupan B. Raaka B. M. Toth M. Gershengorn M. C. (2009). TRH-receptor-type-2-deficient mice are euthyroid and exhibit increased depression and reduced anxiety phenotypes. Neuropsychopharmacol 34, 1601–1608. 10.1038/npp.2008.217 PMC2669701 19078951

[B203] Suzuki M. Sugano H. Matsumoto K. Yamamura M. Ishida R. (1990). Synthesis and central nervous system actions of thyrotropin-releasing hormone analog containing a dihydroorotic acid moiety. J. Med. Chem. 33, 2130–2137. 10.1021/jm00170a014 2115588

[B204] Szabo L. Morey R. Palpant N. J. Wang P. L. Afari N. Jiang C. (2015). Statistically based splicing detection reveals neural enrichment and tissue-specific induction of circular RNA during human fetal development. Genome Biol. 16, 126. 10.1186/s13059-015-0690-5 26076956 PMC4506483

[B205] Szirtes T. Kisfaludy L. Palosi E. Szporny L. (1984). Synthesis of thyrotropin-releasing hormone analogs. 1. Complete dissociation of central nervous system effects from thyrotropin-releasing activity. J. Med. Chem. 27, 741–745. 10.1021/jm00372a006 6429332

[B206] Szirtes T. Kisfaludy L. Palosi E. Szporny L. (1986). Synthesis of thyrotropin-releasing hormone analogs. 2. Tripeptides structurally greatly different from TRH with high central nervous system activity. J. Med. Chem. 29, 1654–1658. 10.1021/jm00159a015 3091831

[B207] Taché Y. Yang H. Miampamba M. Martinez V. Yuan P. Q. (2006). Role of brainstem TRH/TRH-R1 receptors in the vagal gastric cholinergic response to various stimuli including sham-feeding. Autonomic Neurosci. 125, 42–52. 10.1016/j.autneu.2006.01.014 PMC8086327 16520096

[B208] Tache Y. (2012). Brainstem neuropeptides and vagal protection of the gastric mucosal against injury: role of prostaglandins, nitric oxide and calcitonin-gene related peptide in capsaicin afferents. CMC 19, 35–42. 10.2174/092986712803414097 PMC3694172 22300074

[B209] Takeuchi Y. Takano T. Abe J. Takikita S. Ohno M. (2001). Thyrotropin-releasing hormone: role in the treatment of West syndrome and related epileptic encephalopathies. Brain Dev. 23, 662–667. 10.1016/S0387-7604(01)00303-5 11701274

[B210] Tang T. Li L. Tang J. Li Y. Lin W. Y. Martin F. (2010). A mouse knockout library for secreted and transmembrane proteins. Nat. Biotechnol. 28, 749–755. 10.1038/nbt.1644 20562862

[B211] Taylor W. L. Dixon J. E. (1976). The inhibition of thyrotropin-releasing hormone deamidation in porcine hypothalamic tissues. Biochim. Biophys. Acta (BBA) Gen. Subj. 444, 428–434. 10.1016/0304-4165(76)90386-X 822879

[B212] Tenorio-Laranga J. Venäläinen J. I. Männistö P. T. García-Horsman J. A. (2008). Characterization of membrane-bound prolyl endopeptidase from brain: Membrane-bound prolyl oligopeptidase: mPOP. FEBS J. 275, 4415–4427. 10.1111/j.1742-4658.2008.06587.x 18657187

[B213] Terauchi Y. Ishikawa S. Oida S. Negoro T. Kagemoto A. Sekine Y. (1988). Determination of L-6-keto-piperidine-2-carbonyl-L-leucyl-L-proline amide (RGH-2202), a new analog of thyrotropin-releasing hormone, in plasma by radioimmunoassay. J. Pharmacobio. Dynamics 11, 459–464. 10.1248/bpb1978.11.459 3139860

[B214] Thirunarayanan N. Nir E. A. Raaka B. M. Gershengorn M. C. (2013). Thyrotropin-Releasing Hormone Receptor Type 1 (TRH-R1), not TRH-R2, Primarily Mediates Taltirelin Actions in the CNS of Mice. Neuropsychopharmacol 38, 950–956. 10.1038/npp.2012.256 PMC3629383 23303050

[B215] Thompson B. L. Rosen J. B. (2000). Effects of TRH on acoustic startle, conditioned fear and active avoidance in rats. Neuropeptides 34, 38–44. 10.1054/npep.1999.0785 10688967

[B216] Torres H. Charli J. Gonzaleznoriega A. Vargas M. Joseph-Bravo P. (1986). Subcellular distribution of the enzymes degrading thyrotropin releasing hormone and metabolites in rat brain. Neurochem. Int. 9, 103–110. 10.1016/0197-0186(86)90038-0 20493107

[B217] Traube T. Shokhen M. Albeck A. (2014). A new method for filtering of reactive “warheads” of transition-state analog protease inhibitors. Eur. J. Med. Chem. 77, 134–138. 10.1016/j.ejmech.2014.02.059 24631732

[B218] Uhlén M. Fagerberg L. Hallstrom B. M. Lindskog C. Oksvold P. Mardinoglu A. (2015). Tissue-based map of the human proteome. Science 347, 1260419–1260419. 10.1126/science.1260419 25613900

[B219] Urayama A. Yamada S. Hirano K. Deguchi Y. Kimura R. (2001). Brain receptor binding characteristics and pharmacokinetic-pharmacodynamic analysis of thyrotropin-releasing hormone analogues. Life Sci. 70, 647–657. 10.1016/S0024-3205(01)01445-X 11833715

[B220] Urayama A. Yamada S. Kimura R. Zhang J. Watanabe Y. (2002). Neuroprotective effect and brain receptor binding of taltirelin, a novel thyrotropin-releasing hormone (TRH) analogue, in transient forebrain ischemia of C57BL/6J mice. Life Sci. 72, 601–607. 10.1016/S0024-3205(02)02268-3 12467901

[B221] Vargas M. Mendez M. Cisneros M. Joseph-Bravo P. Charli J. L. (1987). Regional distribution of the membrane-bound pyroglutamate amino peptidase-degrading thyrotropin-releasing hormone in rat brain. Neurosci. Lett. 79, 311–314. 10.1016/0304-3940(87)90450-2 2889173

[B222] Vargas M. A. Cisneros M. Herrera J. Joseph-Bravo P. Charli J.-L. (1992a). Regional distribution of pyroglutamyl peptidase II in rabbit brain, spinal cord, and organs. Peptides 13, 255–260. 10.1016/0196-9781(92)90105-C 1357633

[B223] Vargas M. A. Herrera J. Uribe R. M. Charli J.-L. Joseph-Bravo P. (1992b). Ontogenesis of pyroglutamyl peptidase II activity in rat brain, adenohypophysis and pancreas. Dev. Brain Res. 66, 251–256. 10.1016/0165-3806(92)90087-D 1351427

[B224] Veronesi M. C. Kubek D. J. Kubek M. J. (2007). Intranasal delivery of a Thyrotropin-Releasing Hormone analog attenuates seizures in the amygdala-kindled rat. Epilepsia 48, 2280–2286. 10.1111/j.1528-1167.2007.01218.x 17651414

[B225] Vogel R. A. Frye G. D. Wilson J. H. Kuhn C. M. Mailman R. B. Mueller R. A. (1980). Attenuation of the effect of punishment by thyrotropin-releasing hormone: comparisons with chlordiazepoxide. J. Pharmacol. Exp. Ther. 212, 153–161. 6243358

[B226] Wang W. Gershengorn M. C. (1999). Rat TRH receptor type 2 exhibits higher basal signaling activity than TRH receptor type 1. Endocrinology 140, 4916–4919. 10.1210/endo.140.10.7159 10499553

[B227] Wilk S. Wilk E. (1989). Pyroglutamyl peptidase II, a thyrotropin releasing hormone degrading enzyme: purification and specificity studies of the rabbit brain enzyme. Neurochem. Int. 15, 81–89. 10.1016/0197-0186(89)90079-X 20504468

[B228] Wilk S. (1983). Prolyl endopeptidase. Life Sci. 33, 2149–2157. 10.1016/0024-3205(83)90285-0 6358755

[B229] Williams R. S. B. Eames M. Ryves W. J. Viggars J. Harwood A. J. (1999). Loss of a prolyl oligopeptidase confers resistance to lithium by elevation of inositol (1,4,5) trisphosphate. EMBO J. 18, 2734–2745. 10.1093/emboj/18.10.2734 10329620 PMC1171355

[B230] Yamada M. Saga Y. Shibusawa N. Hirato J. Murakami M. Iwasaki T. (1997). Tertiary hypothyroidism and hyperglycemia in mice with targeted disruption of the thyrotropin-releasing hormone gene. Proc. Natl. Acad. Sci. 94, 10862–10867. 10.1073/pnas.94.20.10862 9380725 PMC23510

[B231] Yamada M. Shibusawa N. Hashida T. Satoh T. Monden T. Prasad C. (1999). Abundance of Cyclo (His-Pro)-like immunoreactivity in the brain of TRH-deficient mice. Endocrinology 140, 538–541. 10.1210/endo.140.1.6607 9886867

[B232] Yamada M. Shibusawa N. Hashida T. Ozawa A. Monden T. Satoh T. (2000). Expression of Thyrotropin-Releasing Hormone (TRH) receptor subtype 1 in mouse pancreatic islets and HIT-T15, an insulin-secreting clonal β cell line. Life Sci. 66, 1119–1125. 10.1016/S0024-3205(00)00415-X 10737362

[B233] Yamada M. Shibusawa N. Ishii S. Horiguchi K. Umezawa R. Hashimoto K. (2006). Prolactin secretion in mice with Thyrotropin-Releasing Hormone deficiency. Endocrinology 147, 2591–2596. 10.1210/en.2005-1326 16484326

[B234] Yamamoto M. Shimizu M. (1987). Effects of a new TRH analogue, YM-14673 on the central nervous system. Naunyn-Schmiedeberg's Arch. Pharmacol. 336, 561–565. 10.1007/BF00169314 3125485

[B235] Yamamoto H. Morise K. Kusugami K. Furusawa A. Konagaya T. Nishio Y. (1996). Abnormal neuropeptide concentration in rectal mucosa of patients with inflammatory bowel disease. J. Gastroenterol. 31, 525–532. 10.1007/BF02355052 8844473

[B236] Yamamura M. Kinoshita K. Nakagawa H. Tanaka T. Maeda K. Ishida R. (1990). Pharmacological study of TA-0910, a new thyrotropin-releasing hormone (TRH) analog. (I). Effects on the central nervous system by oral administration. Jpn. J. Pharmacol. 53, 451–461. 10.1254/jjp.53.451 2120494

[B237] Yamamura M. Kinoshita K. Nakagawa H. Ishida R. (1991a). Pharmacological study of TA-0910, a new thyrotropin-releasing hormone (TRH) analog (II): Involvement of the DA system in the locomotor stimulating action of TA-0910. Jpn. J. Pharmacol. 55, 57–68. 10.1254/jjp.55.57 1904114

[B238] Yamamura M. Kinoshita K. Nakagawa H. Ishida R. (1991b). Pharmacological study of TA-0910, a new thyrotropin-releasing hormone (TRH) analog (III): Inhibition of pentobarbital anesthesia. Jpn. J. Pharmacol. 55, 69–80. 10.1254/jjp.55.69 1904115

[B239] Yamaoka T. Itakura M. (1999). Development of pancreatic islets (review). Int. J. Mol. Med. 247–261. 10.3892/ijmm.3.3.247 10028048

[B240] Yang H. Taché Y. Ohning G. Go V. L. W. (2002). Activation of raphe pallidus neurons increases insulin through medullary Thyrotropin-Releasing Hormone (TRH)-vagal pathways. Pancreas 25, 301–307. 10.1097/00006676-200210000-00014 12370543

[B241] Yang H. Nyby M. D. Ao Y. Chen A. Adelson D. W. Smutko V. (2012). Role of brainstem thyrotropin-releasing hormone-triggered sympathetic overactivation in cardiovascular mortality in type 2 diabetic Goto–Kakizaki rats. Hypertens. Res. 35, 157–165. 10.1038/hr.2011.154 21900943

[B242] Yokohama S. Yoshioka T. Yamashita K. Kitamori N. (1984). Intestinal absorption mechanisms of thyrotropin-releasing hormone. J. Pharmacobio. Dynamics 7, 445–451. 10.1248/bpb1978.7.445 6436461

[B243] Yoo S. Cha D. Kim S. Jiang L. Adebesin M. Wolfe A. (2019). Ablation of tanycytes of the arcuate nucleus and median eminence increases visceral adiposity and decreases insulin sensitivity in male mice. bioRxiv. 10.1101/637587

[B244] Yu Y. Zhao C. Su Z. Wang C. Fuscoe J. C. Tong W. (2014). Comprehensive RNA-Seq transcriptomic profiling across 11 organs, 4 ages, and 2 sexes of Fischer 344 rats. Sci. Data 1, 140013. 10.1038/sdata.2014.13 25977771 PMC4381750

[B245] Zacur H. A. Genadry R. Rock J. A. King T. M. Smith B. R. Foster G. V. (1985). Thyrotropin-Releasing Hormone-Induced contraction of urethral and vaginal muscle. J. Clin. Endocrinol. Metab. 61, 787–789. 10.1210/jcem-61-4-787 3928678

[B246] Zada M. H. Kubek M. Khan W. Kumar A. Domb A. (2019). Dispersible hydrolytically sensitive nanoparticles for nasal delivery of thyrotropin releasing hormone (TRH). J. Control Release 295, 278–289. 10.1016/j.jconrel.2018.12.050 30610951

[B247] Zafirov D. H. Cooke H. J. Wood J. D. (1991). Thyrotropin-releasing hormone excites submucous neurons an guinea-pig ileum. Eur. J. Pharmacol. 204, 109–112. 10.1016/0014-2999(91)90843-F 1687122

[B248] Zarif H. Petit-Paitel A. Heurteaux C. Chabry J. Guyon A. (2016). TRH modulates glutamatergic synaptic inputs on CA1 neurons of the mouse hippocampus in a biphasic manner. Neuropharmacology 110, 69–81. 10.1016/j.neuropharm.2016.04.004 27060411

[B249] Zeisel A. Hochgerner H. Lönnerberg P. Johnsson A. Memic F. van der Zwan J. (2018). Molecular architecture of the mouse nervous system. Cell 174, 999–1014.e22. 10.1016/j.cell.2018.06.021 30096314 PMC6086934

[B250] Zeng H. Schimpf B. A. Rohde A. D. Pavlova M. N. Gragerov A. Bergmann J. E. (2007). Thyrotropin-Releasing Hormone receptor 1-deficient mice display increased depression and anxiety-like behavior. Mol. Endocrinol. 21, 2795–2804. 10.1210/me.2007-0048 17666589

[B251] Zhu Q.-S. Rosenblatt K. Huang K.-L. Lahat G. Brobey R. Bolshakov S. (2011). Vimentin is a novel AKT1 target mediating motility and invasion. Oncogene 30, 457–470. 10.1038/onc.2010.421 20856200 PMC3010301

[B252] Zhuan B. Lu Y. Chen Q. Zhao X. Li P. Yuan Q. (2019). Overexpression of the long noncoding RNA TRHDE-AS1 inhibits the progression of lung cancer via the miRNA-103/KLF4 axis. J. Cell Biochem. 120, 17616–17624. 10.1002/jcb.29029 31119790

